# A decrease in *Flavonifractor plautii* and its product, phytosphingosine, predisposes individuals with phlegm-dampness constitution to metabolic disorders

**DOI:** 10.1038/s41421-025-00789-x

**Published:** 2025-03-17

**Authors:** Lingru Li, Tianxing Li, Xue Liang, Linghui Zhu, Yini Fang, Ling Dong, Yi Zheng, Xiaoxue Xu, Mingrui Li, Tianqi Cai, Fufangyu Zhao, Meiling Xin, Mingyan Shao, Yuanyuan Guan, Meiyi Liu, Fangli Li, Chenhong Zhang, Qi Wang, Wenlong Sun, Yanfei Zheng

**Affiliations:** 1 National Institute of Traditional Chinese Medicine Constitution and Preventive Treatment of Diseases, Beijing University of Chinese Medicine, Beijing, China; 2 School of Life Sciences and Medicine, Shandong University of Technology, Zibo, Shandong China; 3 Institute of Basic Theory for Chinese Medicine, China Academy of Chinese Medical Sciences, Beijing, China; 4 Beijing University of Chinese Medicine Affiliated Shenzhen Hospital, Shenzhen, Guangdong China; 5 State Key Laboratory of Microbial Metabolism, School of Life Sciences and Biotechnology, Shanghai Jiao Tong University, Shanghai, China

**Keywords:** Mechanisms of disease, Genomic analysis

## Abstract

According to traditional Chinese medicine (TCM) constitutional theory, individuals with phlegm-dampness constitution (PDC) are at increased risk for metabolic disorders. Previous studies have indicated that PDC individuals exhibit gene expression changes associated with metabolic disorders, even individuals with normal metabolic indices. However, the biological mechanisms underlying these changes remain unclear. The gut microbiota has recently emerged as a promising avenue for elucidating TCM principles. Here, we revealed that individuals with PDC have distinct gut microbiota and serum metabolite profiles. A decrease in phytosphingosine was associated with increased PDC scores and metabolic disorder severity. Subsequent experiments demonstrated that *Flavonifractor plautii* can biosynthesize phytosphingosine, which was also negatively correlated with the PDC score. Interestingly, both *F. plautii* and phytosphingosine levels decreased in PDC subjects with normal metabolic indices. Fecal transplantation from these individuals accelerated the development of metabolic disorders in mice. However, supplementation with *F. plautii* and phytosphingosine ameliorated metabolic disorders by increasing phytosphingosine levels in the gut‒hepatic axis. Mechanistic investigations confirmed that phytosphingosine can directly bind to hepatic peroxisome proliferator-activated receptor α (PPARα) and activate its nuclear transcription activity, thereby regulating downstream gene expression related to glucose‒lipid metabolism. Our research indicates that the decrease in *F. plautii* and its product, phytosphingosine, contributes to gene expression changes related to metabolic disorders in PDC individuals and increases their susceptibility to metabolic disorders. These findings suggest that diagnosing PDC may be beneficial for identifying at-risk populations among apparently healthy individuals, thereby advancing the broader field of metabolic disorder prevention and TCM integration.

## Introduction

Metabolic disorders represent a group of disorders that include various interrelated pathological conditions, such as overweight/obesity, hyperlipidemia, diabetes, hypertension, and hyperuricemia^1^. Over the last two decades, due to diet and lifestyle changes, the global incidence of these diseases has reached a high level^2–5^, which significantly increases the morbidity and mortality rates of cardiovascular diseases. Early diagnosis and prevention of metabolic disorders are important for preventing and blocking disease processes and reducing the public health burden^6–8^. At present, according to the consensus of experts, overweight, prediabetes, and borderline hyperlipidemia are still important primary or secondary prevention indicators^9^. However, abnormal metabolic indices indicate long-lasting changes in the physiological characteristics of the whole body^10^. There is an urgent need for a method that can identify precursor changes before metabolic diseases occur .

Traditional Chinese medicine (TCM) is a type of medicine that originated in China. Holism is its fundamental principle, and the preventive treatment of disease represents one of its advantages. TCM obtains systemic characteristics through four diagnostic methods (sight, hearing, questioning, and pulse) to predict changes in the pathogenesis of the body rather than relying on a single clinical indicator; thus, precursor changes are potentially identified before the clinical indicators change^11^. With the development of TCM, diagnosis is no longer dependent on the characteristics collected using the four diagnostic methods; rather, it has gradually changed to quantification, objectification and standardization. The term “constitution” refers to the unique TCM “type“^12^. On the basis of the literature and an epidemiological study of the Han Chinese population, we have defined nine constitutional groups in the population, namely, a balanced constitution (BC) group and eight unbalanced constitution groups. We have also formulated classification criteria, such as the 1–5 graded scoring methods, for the nine constitutional groups. This scoring system has been issued as the industry standard of TCM (version: ZYYXH/T157-2009)^13^. BC individuals account for less than 30% of the population^14^. The clinical phenotypic features of individuals with BC include high energy, loud voices, stable moods, adaptability to external changes, good sleep quality, good memory, and no other unbalanced constitutional features. These individuals are at a low risk for various diseases. People with unbalanced constitutions always suffer from or face the risk of various diseases^15^. The phlegm-dampness constitution (PDC) is an unbalanced constitution. Individuals with PDCs account for nearly 10% of the population^14^. The clinical phenotypic features of PDC are as follows: heavy body, stuffy chest, full abdomen, loose and soft abdominal skin, greasy forehead, upper eyelid edema, phlegm obstruction in the throat, sticky mouth, and thick tongue coating^16^. The PDC scoring system from the constitutional industry standard can be used to diagnose PDC. Meta-analyses of epidemiological and clinical investigations confirmed that PDC subjects are at high risk of metabolic disorders such as obesity, hyperlipidemia, hypertension and diabetes^17–20^. Previous studies have shown that PDC individuals, even those with normal metabolic indices, exhibit gene expression changes related to metabolic disorders^21^. However, the biological mechanism of these precursor changes is still unclear.

From the perspective of TCM, people’s various tissues and organs are not independent but rather interconnected and together influence the whole-body physiological state and pathological changes. Recently, many studies have shown that the gut microbiota can connect various tissues and organs, which is an important biological basis for affecting systemic physiological and pathological states^22^. Thus, the gut microbiota has emerged as a new avenue for elucidating TCM principles^23^. The gut microbiota participates in host metabolism via its derived metabolites. Previous studies have shown that microbial metabolites play a critical role in the development of metabolic disorders^24–28^. Our team and others have reported that individuals with PDC have a distinctive gut microbial structure^29–31^. However, it remains to be revealed whether gut bacteria and related metabolites contribute to gene expression changes related to metabolic disorders in PDC individuals.

Here, we applied 16S rRNA gene sequencing and metabolic analyses to investigate how the gut microbiota and related metabolite characteristics contribute to the susceptibility of PDC to metabolic disorders. Through comparisons with BC subjects, correlation analysis and a series of follow-up experiments, such as metagenome sequencing and qPCR verification of bacteria, in vitro bacterial anaerobic culture experiments, in vivo bacteria-colonized germ-free mouse experiments, and metabolite-targeted monitoring of serum and culture media, we highlight *Flavonifractor plautii* and its product, phytosphingosine, which are negatively correlated with PDC scores. Both *F. plautii* and phytosphingosine decreased in PDC individuals with normal metabolic indices. Fecal transplantation from these individuals accelerated the development of metabolic disorders in mice. Supplementation with *F. plautii* and phytosphingosine improved metabolic disorders induced by fecal transplantation from PDC subjects with normal metabolic indices and a high-fat diet (HFD) by increasing phytosphingosine levels in the gut‒hepatic axis. Mechanistic investigations confirmed that phytosphingosine can directly bind to hepatic PPARα and activate its nuclear transcription activity, thereby regulating downstream gene expression. In particular, these findings provide an explanation for the gene expression changes related to metabolic disorders in PDC individuals and may be beneficial for the early detection and intervention of metabolic disorders.

## Results

### Clinical characteristics of the study population

**Fig. 1:**
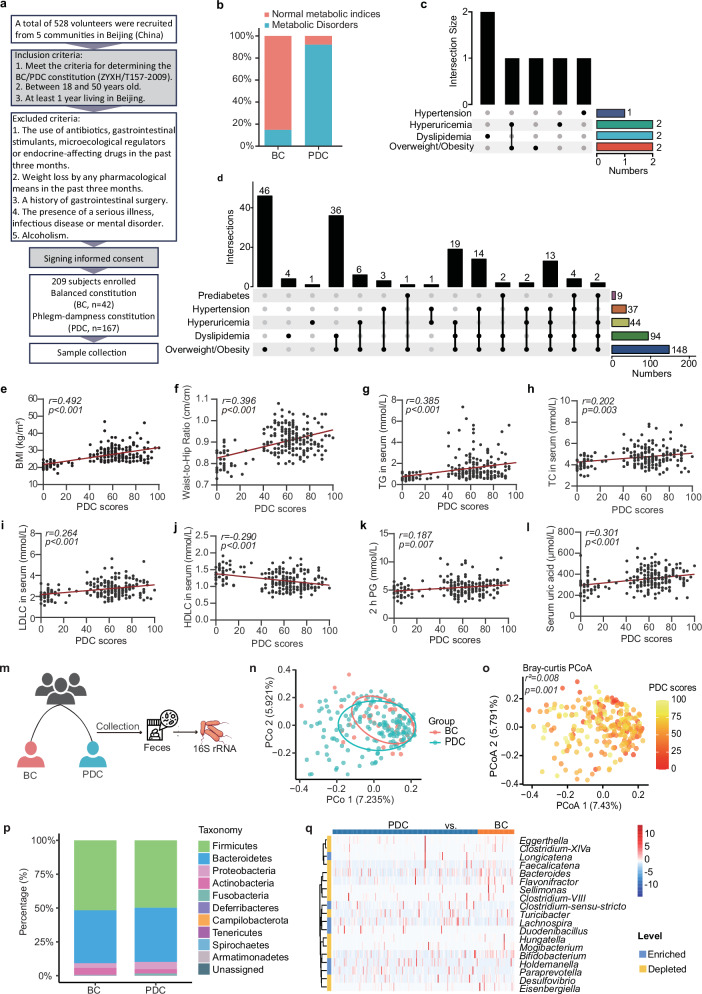
Diagnostic indices for PDC individuals. The PDC scores were significantly correlated with metabolic indices, and PDC subjects had different gut microbial structures than BC subjects did.**a** Schematic representation of the clinical sample collection process. A total of 209 subjects, including 167 PDC subjects and 42 BC subjects, were enrolled. The inclusion criteria were as follows: 1. met the criteria for determining the BC/PDC constitution (ZYXH/T157-2009), 2. aged between 18 and 50 years, and 3. had at least 1 year of living in Beijing. The exclusion criteria were as follows: 1. the use of antibiotics, gastrointestinal stimulants, microecological regulators or endocrine-affecting drugs in the past three months; 2. weight loss by any pharmacological means in the past three months; 3. a history of gastrointestinal surgery; 4. the presence of a serious illness, infectious disease or mental disorder; or 5. alcoholism. In this cohort, we characterized the gut microbiome and serum metabolome and identified microbial and metabolic characteristics. **b** Total disease and health status of the PDC group and BC group. The blue color represents the subjects with normal metabolic indices; the orange color represents the subjects with metabolic disorders. **c** Details of the disease and health status of the subjects in the BC group. **d** Detailed disease and health status of the PDC group. **e**–**l** Spearman correlations (two-tailed Spearman’s rank test) between PDC scores and BMI (**e**), WHR (**f**), serum TG levels (**g**), serum TC levels (**h**), serum LDL-C levels (**i**), serum HDLC levels (**j**), 2 h PG (**k**), and serum uric acid levels (**l**). **m** Procedure for collecting human fecal samples. Fecal samples were collected, and the V3‒V4 region of the 16S rRNA gene was sequenced to identify microbial characteristics. **n** Principal coordinate analysis (PCoA) of the gut microbiota calculated from the Bray‒Curtis distance in the BC group and PDC group. p values were calculated with adonis via 6000 permutations. **o** The same PCoA plot (**n**), colored by PDC score. Gut microbial compositions of the BC group and PDC group at the phylum level (**p**) or the genus level (**q**). BC balanced constitution, PDC phlegm-dampness constitution, BMI body mass index, WHR waist‒to‒hip ratio, TG triglyceride, TC total cholesterol, LDLC low-density lipoprotein cholesterol, HDLC high-density lipoprotein cholesterol; 2 h PG, 2 h postprandial blood glucose.

A total of 209 subjects (167 PDC subjects and 42 BC subjects) were selected according to the TCM constitution determination industry criteria (Version: ZYYXH/T157-2009) from a pool of 528 volunteers in 5 communities in Beijing (China) over a period of 5 months (Jan–Jun 2019) (Fig. 1a). To understand the clinical characteristics of PDC subjects compared with those of BC subjects we collected comprehensive clinical metadata, including detailed demographic data, as well as clinical data such as weight, lipid, blood glucose, uric acid and blood pressure data. Compared with the BC group, the PDC group presented elevated body weight, body mass index (BMI), waist‒to‒hip ratio (WHR), triglyceride (TG), total cholesterol (TC), low-density lipoprotein cholesterol (LDLC), fasting insulin, uric acid, systolic blood pressure (SBP), and diastolic blood pressure (DBP) (Supplementary Table S1). According to the clinical diagnostic criteria for metabolic disorders, only ~ 14.29% of the BC subjects exhibited metabolic disorders (6 subjects, Fig. 1b, c). Approximately 92.22% of the PDC subjects had one or more metabolic disorders (154 subjects), with overweight/obesity accounting for the highest proportion (148 subjects), followed by dyslipidemia (94 subjects) and hyperuricemia (44 subjects) (Fig. 1b, d). Furthermore, Spearman’s correlation analysis revealed that the PDC scores were significantly positively correlated with BMI (*r* = 0.492, *p* < 0.001), WHR (*r* = 0.396, *p* < 0.001), serum TG (*r* = 0.385, *p* < 0.001), serum TC (*r* = 0.202, *p* = 0.003), serum LDLC (*r* = 0.264, *p* < 0.001), serum high-density lipoprotein cholesterol (HDLC) (*r* = –0.290, *p* < 0.001), serum apolipoprotein A1 (ApoA1) (*r* = –0.177, *p* = 0.01), serum apolipoprotein B (ApoB) (*r* = 0.223, *p* = 0.001), fasting blood glucose (*r* = 0.097, *p* = 0.162), 2 h postprandial blood glucose (2 h-PG) (*r* = 0.187, *p* = 0.007), fasting insulin (*r* = 0.399, *p* < 0.001), serum uric acid (*r* = 0.301, *p* < 0.001), SBP (*r* = 0.244, *p* < 0.001), and DBP (*r* = 0.279, *p* < 0.001) (Fig. 1e–l and Supplementary Fig. S1a–f). Collectively, these results suggest that the PDC score can represent the degree of metabolic disorders.

### Shifts in the gut microbiota in the PDC group and BC group

To characterize the microbiome profile of the PDC group, we collected fecal samples from all the subjects in the clinical cohort for gut microbiota analysis (Fig. 1m). We investigated the changes in the gut microbiome between the PDC group and BC group by sequencing the fecal 16S rRNA V3‒V4 region. No significant differences in the richness or diversity of the gut microbiota were found between the PDC group and the BC group (Supplementary Fig. S2a, b). Principal coordinate analysis (PCoA) calculated from the Bray‒Curtis distance was performed to display the variation in community composition, which significantly differed between the PDC group and the BC group (*p* = 0.009; Fig. 1n). Notably, we found that the PDC scores can explain the differences along the first principal coordinate (*p* = 0.001; Fig. 1o). The gut microbial community consisted of 187 genera belonging to 10 phyla. At both the phylum and genus levels, the microbial composition of the PDC group differed from that of the BC group (Fig. 1p, q and Supplementary Fig. S3). To further identify which bacterial taxa were distinct between the PDC group and BC group, we compared the mean relative abundances of more than 0.1% of the genera. Nineteen genera showed significant differences, and *Flavonifractor*, *Bifidobacterium*, *Faecalicatena*, *Eggerthella*, and *Bacteroides* (the top 5 genera differed the most) were depleted in the PDC group compared with those in the BC group (Fig. 1q).

### Shifts in the serum metabolic profiles of the PDC group and BC group

**Fig. 2:**
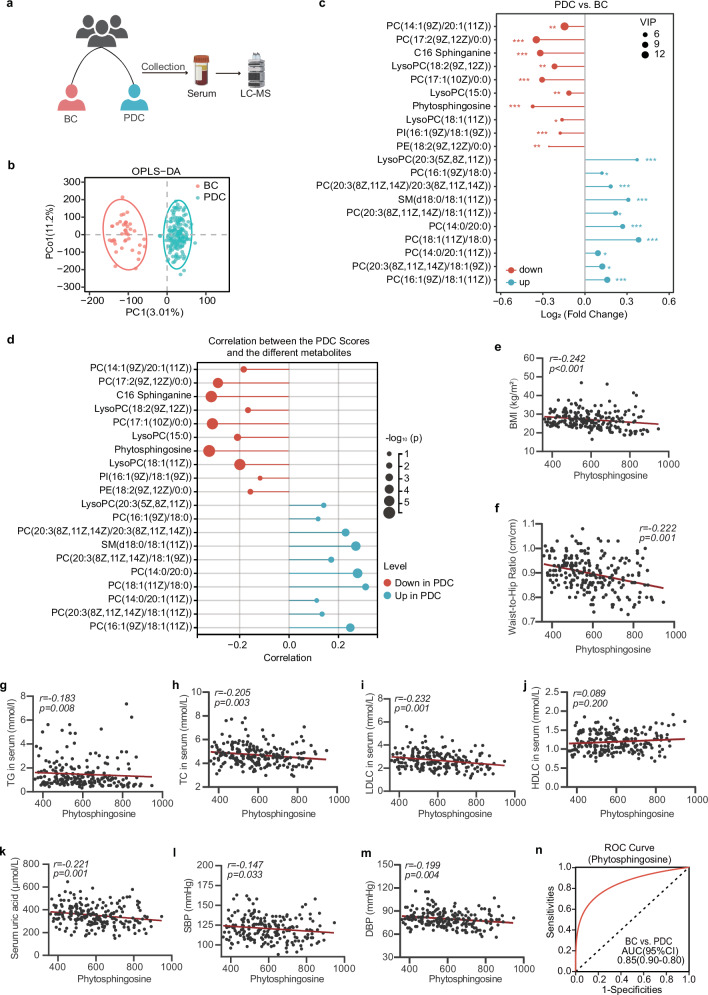
PDC subjects had different serum metabolite structures than BC subjects did, and phytosphingosine was significantly correlated with PDC scores and metabolic indices. **a** Procedure for collecting human serum samples. Serum samples were collected, and untargeted metabolomics was performed to identify metabolic characteristics. **b** OPLS–DA plot of the serum metabolites in the BC group and PDC group. The p values of the model are calculated via 1000 permutations. **c** The 10 most significantly altered metabolites of the PDC group with the highest VIP values among the up- and downregulated metabolites in comparison with those of the BC group. **d** Correlated bubble diagram of PDC scores with serum metabolic profiles. Red and blue indicate enrichment in BC and PDC individuals, respectively. Spearman correlations (two-tailed Spearman’s rank test) were performed. **e**–**m** Spearman correlations between serum phytosphingosine abundance and BMI (**e**), WHR (**f**), serum TG levels (**g**), serum TC levels (**h**), serum LDLC levels (**i**), serum HDLC levels (**j**), serum uric acid levels (**k**), SBP (**l**), and DBP (**m**). **n** ROC curve of the random forest model using phytosphingosine to distinguish PDC subjects from BC subjects. BC balanced constitution, PDC phlegm-dampness constitution, BMI body mass index, WHR waist‒to‒hip ratio, TG triglyceride, TC total cholesterol, LDLC low-density lipoprotein cholesterol, HDLC high-density lipoprotein cholesterol, SBP systolic blood pressure, DBP diastolic blood pressure.

To characterize the metabolic profile of the PDC group, untargeted metabolome profiling was performed on fasting serum samples from all the subjects via liquid chromatography–tandem mass spectrometry (LC–MS/MS) (Fig. 2a). A total of 1822 metabolites were captured via untargeted metabolomics, and significant differences were detected between the PDC group and the BC group via orthogonal partial least squares discriminant analysis (OPLS‒DA) (Fig. 2b). The volcano plots revealed that a total of 123 serum metabolites were changed in the PDC group compared with those in the BC group, of which 70 metabolites were significantly upregulated and 53 metabolites were significantly downregulated (screening criteria were *p* < 0.05 and variable importance in projection (VIP) > 1.0) (Supplementary Fig. S4). We focused on the 10 differentially abundant metabolites with the largest VIP values, either upregulated or downregulated, in the PDC group compared with those in the BC group. The results revealed that the differentially abundant metabolites included mainly phytosphingosine, PCs, LysoPCs, and C16 sphinganine (Fig. 2c).

### A decrease in phytosphingosine was associated with higher PDC scores

Next, we analyzed the correlation between the differentially abundant metabolites and the PDC scores. We focused on the metabolite phytosphingosine, which was most negatively correlated with the PDC scores (Fig. 2d). Moreover, the level of phytosphingosine was also significantly correlated with metabolic indices (Fig. 2e–m and Supplementary Fig. S5) related to obesity (BMI (*r* = −0.242, *p* < 0.001), WHR (*r* = −0.222, *p* = 0.001)), lipid metabolism (serum TG (*r* = −0.183, *p* = 0.008), TC (*r* = −0.205, *p* = 0.003), LDLC (*r* = −0.232, *p* = 0.001), and uric acid (*r* = −0.221, *p* = 0.001)). We next developed a machine learning classifier based on a random forest algorithm to distinguish individuals with either PDC from those with BC via the value of phytosphingosine. Receiver operating characteristic curve analysis revealed that the model had good prediction power, with a mean cross-validation area under the curve (AUC) of 0.85 for differentiating PDC subjects and BC subjects (Fig. 2n). These data indicated that a decrease in phytosphingosine was associated with higher PDC scores, which might increase susceptibility to metabolic disorders in PDC individuals.

### *F. plautii* biosynthesized phytosphingosine, which was negatively correlated with the PDC score

**Fig. 3:**
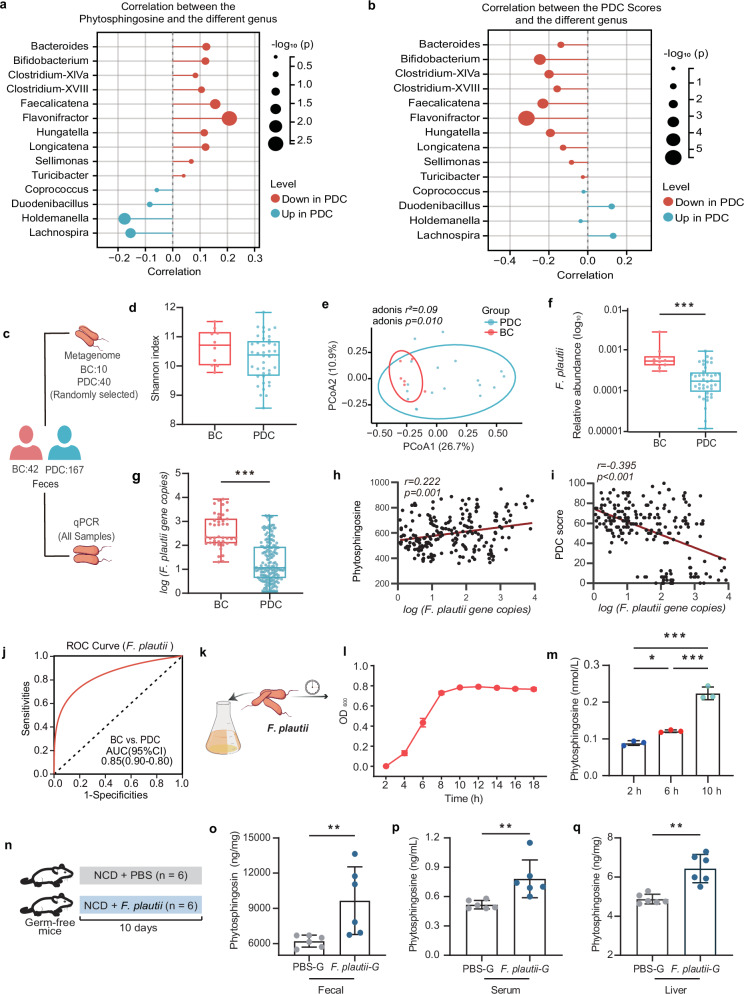
*F. plautii*, which was significantly correlated with the phytosphingosine and PDC scores, can biosynthesize phytosphingosine in vitro and in germ-free mice. **a** Correlated bubble diagram of phytosphingosine with various genera. **b** Correlated bubble diagram of PDC scores with differential genera. Red and blue indicate enrichment in BC and PDC individuals, respectively. Spearman correlations (two-tailed Spearman’s rank test) were performed. **c** Procedure for macrogenome sequencing and qPCR. Fecal samples were collected from all the subjects. Fecal samples from 40 PDC subjects and 10 age- and sex-matched BC subjects were subjected to metagenomic analysis to identify microbial characteristics. Fecal samples from all the subjects were subjected to qPCR to measure the levels of *F. plautii*. **d**, **e** Shannon indices (**d**) and PCoA plot (**e**) calculated from the Bray‒Curtis distance in 40 PDC subjects and 10 age- and sex-matched BC subjects. The differences were determined by the Wilcoxon rank-sum test. *p* values were calculated with adonis via 6000 permutations. **f**, **g** Relative abundance (**f**) and gene copies (**g**) of *F. plautii* in 40 PDC subjects and 10 age- and sex-matched BC subjects. The differences were determined by the Wilcoxon rank-sum test or *t* test. **h,** **i** Spearman correlations (two-tailed Spearman’s rank test) between the gene copies of *F. plautii* and serum phytosphingosine abundance (**h**) and PDC scores (**i**). **j** ROC curve of the RF model in which *F. plautii* was used to distinguish PDC subjects from BC subjects. **k** A schematic diagram of *F. plautii* in vitro culture. *F. plautii was* cultured in modified BHI broth. This experiment was repeated 3 times. **l** Growth curve of *F. plauti*i in BHI broth. **m** Phytosphingosine levels at different growing points of *F. plautii* in BHI broth. Differences among groups were analyzed by one-way ANOVA with Tukey’s post hoc test. **n** Schematic representation of germ-free mice supplemented with *F. plautii* or not (*n* = 6). Eight-week-old GF mice were gavaged with phosphate-buffered saline containing *F. plautii* or lacking *F. plautii* and then maintained on a normal diet for 10 days. **o**–**q** Fecal (**o**), serum (**p**), and liver (**q**) phytosphingosine levels of germ-free mice supplemented with F. plautii or not. BC, balanced constitution; PDC, phlegm-dampness constitution; PBS-G group: the germ-free mice were gavaged with PBS every day; *F. plautii-*G group: the germ-free mice were gavaged with 5 × 10^8^ cfu *F. plautii* every day. The differences (**o**–**q**) were determined via *t*-tests. The data in the bar plot are presented as the means ± SD. *, *p* < 0.05, **, *p* < 0.01, and ***, *p* < 0.001, respectively.

The main source of phytosphingosine in the host is the gut microbiota. Thus, a correlation analysis between phytosphingosine and various genera was performed to explore the microbial source of phytosphingosine. We reported that *Flavonifractor* had the strongest positive correlation with phytosphingosine, suggesting that these bacteria may contribute to phytosphingosine alterations (Fig. 3a). Interestingly, *Flavonifractor* also had the strongest positive correlation with the PDC score (Fig. 3b). These results indicated that *Flavonifractor* was closely related to PDC and might substantially contribute to serum phytosphingosine levels. To further identify which gut bacteria contribute to phytosphingosine at the species level, we selected fecal samples from 40 PDC subjects and 10 age- and sex-matched BC subjects for metagenomic sequencing, and the results of the metagenomic sequencing analysis were verified by qPCR in all the fecal samples (Fig. 3c and Supplementary Table S2). The results revealed no significant differences in species richness or diversity between the PDC group and BC group (Fig. 3d), but significant differences in microbial community composition were detected via PCoA (*p* = 0.010, Fig. 3e), which was consistent with the results of 16S rRNA gene sequencing. Our investigation focused on *Flavonifractor* species. Our findings revealed that *F. plautii* was the major species of *Flavonifractor*. Moreover, there was a significant decrease in the relative abundance of *F. plautii* in the PDC group compared with those in the BC group (*p* < 0.001, Fig. 3f). Furthermore, the results of the qPCR quantification confirmed that the level of *F. plautii* in the PDC group was lower than that in the BC group (*p* < 0.001, Fig. 3g and Supplementary Fig. S6). More importantly, *F. plautii* had a positive correlation with phytosphingosine (*r* = 0.222, *p* = 0.001; Fig. 3h) and a negative correlation with PDC scores (*r* = −0.395, *p* < 0.001; Fig. 3i). Moreover, ROC curve analysis revealed that *F. plautii* was a good predictor for distinguishing PDC subjects from BC subjects (AUC = 0.85, Fig. 3j).

To investigate whether *F. plautii* can biosynthesize phytosphingosine, we performed in vitro experiments in *F. plautii* anaerobic culture and in vivo experiments in *F. plautii*-colonized germ-free mice. In vitro, we conducted a growth curve analysis of cultured *F. plautii* in modified brain heart infusion (BHI) broth (Fig. 3k). Our findings indicated that the organism was capable of producing phytosphingosine within a relatively short period (Fig. 3l, m). In vivo, 8-week-old germ-free (GF) mice were gavaged with phosphate-buffered saline (PBS) containing *F. plautii* or lacking *F. plautii* and then maintained on a normal diet for 10 days (Fig. 3n). Compared with those of the mice that were not colonized with *F. plautii*, the phytosphingosine levels in the feces, serum, and liver of the mice colonized with *F. plautii* were greater (Fig. 3o–q). These data suggested that *F.* plautii could biosynthesize phytosphingosine. *F.* plautii was negatively correlated with the PDC score. Its reduction might increase susceptibility to metabolic disorders in PDC individuals.

**Fig. 4:**
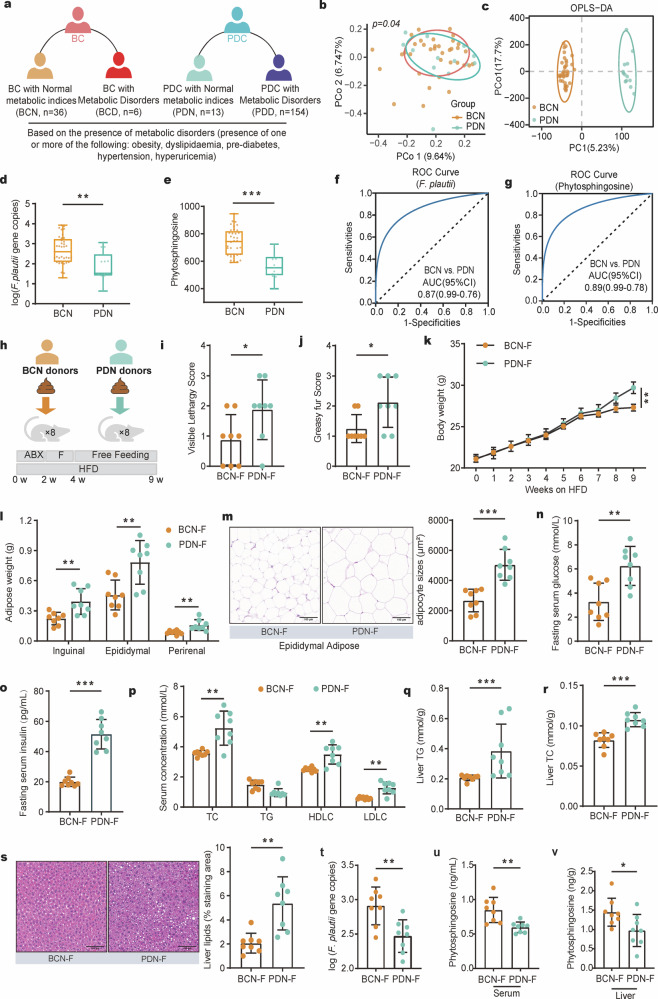
PDC subjects presented lower *F. plautii* and phytosphingosine levels, even those with normal metabolic indices, and fecal transplantation of these subjects accelerated the development of metabolic disorders in mice. **a** Subgrouping schematic representation of the BC group and PDC group. A total of 209 fecal samples, including 167 PDC samples and 42 BC samples, were collected. According to the presence of metabolic disorders (one or more of the following: obesity, dyslipidemia, prediabetes, hypertension, or hyperuricemia), the BC subjects were divided into a BCN (BC subjects with normal metabolic indices) group and a BCD (BC subjects with metabolic disorders) group, and PDC subjects were divided into a PDN (PDC subjects with normal metabolic indices) group and a PDD (PDC subjects with metabolic disorders) group. **b** PCoA of the gut microbiota calculated from the Bray‒Curtis distance in the BCN group and PDN group. *p* values were calculated with adonis via 6000 permutations. **c** OPLS–DA plot of the serum metabolites in the BCN group and PDN group. The *p* values of the model are calculated via 1000 permutations. **d**, **e** Gene copies of *F. plautii* (**d**) and the levels of phytosphingosine (**e**) in the BCN and PDN groups. Differences among groups (**d**, **e**) were analyzed by one-way ANOVA with Tukey’s post hoc test. **f** ROC curve of the RF model using *F. plautii* to distinguish PDN subjects from BCN subjects. **g** ROC curve of the RF model using phytosphingosine to distinguish PDN subjects from BCN subjects. **h** Schematic representation of the fecal transplantation experiment. All the mice were treated with an antibiotic cocktail for two cycles. Following antibiotic treatment, the recipient mice received fecal slurry from the PDN group or BCN group daily for 14 consecutive days and were fed a HFD for 5 weeks. **i**, **j** Visible lethargy scores (**i**) and greasy fur scores (**j**) in the BCN-F group and PDN-F group. **k** Body weight (**k**) and adipose weight (**l**) during the fecal transplantation experimental period. **m** Representative images of adipocyte H&E staining and adipocyte sizes (%) in the BCN-F group and PDN-F group; images were taken at ×40 magnification. Scale bars, 100 μm. *n* = 8 for each group. **n**, **o** Fasting serum glucose (**n**) and serum uric acid (**o**) levels in the BCN-F group and PDN-F group. **p** Serum concentrations of TC, TG, LDLC, and HDLC in the BCN-F group and PDN-F group. **q,** **r** Liver TG (**q**) and liver TC (**r**) levels in the BCN-F group and PDN-F group. **s** Representative images of H&E-stained liver samples (scale bar, 100 μm). Liver lipids (%) in the BCN-F group and PDN-F group. **t** Gene copies of *F. plautii* in the BCN-F group and PDN-F group. **u**, **v** The levels of serum (**u**) and liver (**v**) phytosphingosine in the BCN-F group and PDN-F group. UA, uric acid; TG, triglyceride; TC, total cholesterol. In the BCN-F group, the antibiotic-treated mice received fecal slurry from the BCN group daily for 14 consecutive days and were fed a HFD for 5 weeks; in the PDN-F group, the antibiotic-treated mice received fecal slurry from the PDN group daily for 14 consecutive days and were fed a HFD for 5 weeks. The differences (**i**‒**v**) were determined via *t*-tests. The data in the bar plot are presented as the means ± SD. *, *p* < 0.05, **, *p* < 0.01, and ***, *p* < 0.001, respectively.

### The *F. plautii* and phytosphingosine levels decreased in PDC subjects, including those with normal metabolic indices

Based on the diagnostic criteria of metabolic disorders (one or more of the following: overweight/obesity, dyslipidemia, prediabetes, hypertension, or hyperuricemia), approximately 85.71% of the BC subjects presented normal metabolic indices (BC subjects with normal metabolic indices, BCN, *n* = 36), and 14.29% presented abnormal metabolic indices or metabolic disorders (BC subjects with metabolic disorders, BCD, *n* = 6). In addition, ~ 7.78% of PDC subjects exhibited normal metabolic indices (PDC subjects with normal metabolic indices, PDN, *n* = 13), and 92.22% exhibited abnormal metabolic indices or metabolic disorders (PDC subjects with metabolic disorders, PDD, *n* = 154) (Fig. 4a). These findings support the idea that individuals with BC are at a low risk for various diseases, whereas individuals with PDC are at high risk of metabolic disorders. PCoA calculated from the Bray‒Curtis distance was performed to display the variation in community composition. The results revealed a significant difference between the PDN group and the BCN group (*p* = 0.04, Fig. 4b) but no difference between the PDN group and the PDD group (Supplementary Fig. S7a). The metabolite structure also supported this conclusion (Fig. 4c and Supplementary Fig. S7b). More importantly, the levels of *F. plautii* and phytosphingosine were significantly lower in the PDN group than in the BCN group (Fig. 4d, e). BCN and PDN (AUC = 0.87 or 0.89) can be effectively distinguished via the *F. plautii* or phytosphingosine classifier model (Fig. 4f, g). These data showed that PDC subjects with normal metabolic indices also tend to have metabolic disorders. These individuals have relatively low levels of *F. plautii* and serum phytosphingosine, which might be the biological basis for their susceptibility to metabolic disorders.

### Fecal transplantation from PDC subjects with normal metabolic indices accelerated the development of metabolic disorders in mice

Furthermore, we administered fecal transplantation from the PDN group (PDN-F) and fecal transplantation from the BCN group (BCN-F) to antibiotic-treated mice (Fig. 4h and Supplementary Table S3). At 5 weeks posttransplantation, the visible lethargy scores and greasy fur scores of the PDN-F group were greater than those of the BCN-F group (*p* < 0.05, Fig. 4i, j), indicating that fecal transplantation from the PDC group worsened the PDC phenotypes. In terms of obesity phenotypes, compared with the BCN-F group, the PDN-F group presented significantly greater weight gain (*p* < 0.01, Fig. 4k) with no difference in food intake (*p* > 0.05, Supplementary Fig. S8a). Moreover, the amounts of inguinal fat, epididymal fat, and perirenal fat markedly increased in the PDN-F group (*p* < 0.01, Fig. 4l), and hematoxylin‒eosin (H&E) staining revealed considerably increased adipocyte size in the epididymal fat of the PDN-F group (*p* < 0.001, Fig. 4m). In terms of glucose metabolism phenotypes, compared with those in the BCN-F group, fasting serum glucose (*p* < 0.01, Fig. 4n) levels, fasting serum insulin (*p* < 0.001, Fig. 4o) levels, and homeostasis model assessment — insulin resistance (HOMA-IR, *p* < 0.001, Supplementary Fig. S8b) levels increased. In terms of uric acid metabolism phenotypes, serum uric acid levels were markedly increased in the PDN-F group (*p* < 0.05, Supplementary Fig. S8c). With respect to lipid metabolism phenotypes, dramatically higher serum TC, LDLC, and HDLC levels were observed in the PDN-F group (*p* < 0.01, Fig. 4p). The liver TC (*p* < 0.001) and TG (*p* < 0.001) contents increased markedly, and the liver alanine transaminase (*p* < 0.05, ALT) and aspartate aminotransferase (*p* < 0.001, AST) contents decreased markedly in the PDN-F group (Fig. 4q, r and Supplementary Fig. S8d, e). Moreover, H&E staining of liver tissue revealed a considerable increase in lipid accumulation in the liver tissue of the PDN-F group (Fig. 4s). These results show that multiple metabolic disorders are aggravated by fecal transplantation from PDC subjects with normal metabolic indices. Notably, *F. plautii* demonstrated a similar trend between recipient mice and fecal donor individuals, with a significant decrease in *F. plautii* abundance in the PDN-F group (*p* < 0.01, Fig. 4t). The levels of phytosphingosine in the serum (*p* < 0.01), and liver (*p* < 0.05) of the mice also decreased in the PDN-F group (Fig. 4u, v). These results suggest that the levels of *F. plautii* and phytosphingosine are correlated with the degree of metabolic disorders. In addition, the reduction in *F. plautii* and phytosphingosine serves as a precursor of metabolic disorders, representing one of the biological bases for PDN individuals to be prone to metabolic disorders.

### *F. plautii* alleviates the metabolic disorders induced by a HFD and fecal transplantation of PDC subjects with normal metabolic indices

**Fig. 5:**
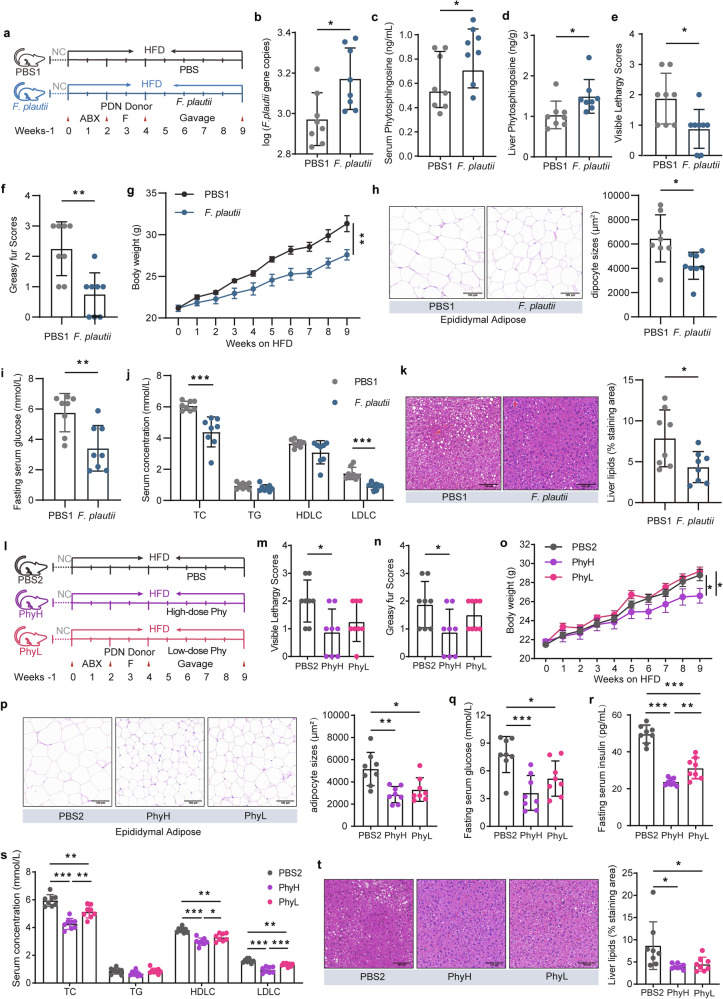
*F. plautii* and phytosphingosine ameliorate metabolic disorders induced by HFD and fecal transplantation in PDC subjects with normal metabolic indices. **a** Schematic representation of the *F. plautii* supplementation experiment. PDN-F mice were treated with PBS (the PBS1 group) as a control, and PDN-F mice were treated with *F. plautii* as the *F. plautii* group for 5 weeks. **b** Gene copy numbers of *F. plautii* in the PBS1 group and *F. plautii* group. **c**, **d** The levels of serum (**c**) and liver (**d**) phytosphingosine in the PBS1 group and *F. plautii* group. **e**, **f** Visible lethargy scores (**e**) and greasy fur scores (**f**) in the PBS1 group and *F. plautii* group. **g** Body weight during the *F. plautii* supplementation experimental periods. **h** Representative images of adipocyte H&E staining and adipocyte sizes (%) in the PBS1 group and *F. plautii* group; images were taken at ×40 magnification. Scale bars: 100 μm. *n* = 8 for each group. **i** Serum glucose levels in the PBS1 group and *F. plautii* group. **j** Serum concentrations of TC, TG, LDLC, and HDLC in the PBS1 group and *F. plautii* group. **k** Representative images of H&E-stained liver samples (scale bar, 100 μm). Liver lipids (%) in the PBS1 group and *F. plautii* group. The differences (**b**–**k**) were determined via a *t*-test. **l** Schematic representation of the phytosphingosine supplementation experiment. PDN-F mice were treated with PBS (PBS1 group) as a control, and PDN-F mice were treated with high or low doses of phytosphingosine (PhyH group or PhyL group, respectively) for 5 weeks. **m**, **n** Visible lethargy scores (**m**) and greasy fur scores (**n**) in the PBS2, PhyH, and PhyL groups. **o** Body weight during the phytosphingosine supplementation experimental periods. **p** Representative images of adipocyte H&E staining and adipocyte sizes (%) in the PBS2, PhyH, and PhyL groups; images were taken at ×40 magnification. Scale bars: 100 μm. *n* = 8 for each group. **q** Serum glucose levels in the PBS2, PhyH, and PhyL groups. **r** Fasting serum insulin in the PBS2, PhyH, and PhyL groups. **s** Serum concentrations of TC, TG, LDLC, and HDLC in the PBS2, PhyH, and PhyL groups. **t** Representative images of H&E-stained liver samples (scale bar, 100 μm) and liver lipids (%) in the PBS2, PhyH, and PhyL groups. Differences among groups (**m**‒**t**) were analyzed by one-way ANOVA with Tukey’s post hoc test. PDN-F mice received fecal slurry from the PDN group daily for 14 consecutive days and were fed a HFD for 5 weeks. In the PBS1 group, the PDN-F mice were gavaged with PBS every day in animal experiment 2. In the *F. plautii* group, PDN-F mice were gavaged with 5 × 10^8^ cfu *F. plautii* every day in the animal experiment. 2. In the PBS2 group, the PDN-F mice were gavaged with PBS every day in animal experiment 3. In the PhyH group, PDN-F mice were gavaged with 50 mg/kg phytosphingosine every day in animal experiment 3. In the PhyL group, PDN-F mice were gavaged with 25 mg/kg phytosphingosine every day in animal experiment 3. *, *p* < 0.05, **, *p* < 0.01, and ***, *p* < 0.001, respectively. The data in the bar plot are presented as the means ± SD.

We used PDN-F mice treated with PBS (PBS1 group) as controls and PDN-F mice treated with *F. plautii* (*F. plautii* group) to investigate the effects of *F. plautii* on metabolic disorders (Fig. 5a). First, the key gut microbiota and metabolite phenotypes of the recipient mice were analyzed via qPCR and LC‒MS/MS. Daily supplementation with *F. plautii* for 5 weeks in PDN-F mice led to a significant increase in the abundance of *F. plautii* in the fecal samples (*p* < 0.05, Fig. 5b and Supplementary Fig. S9a). The levels of phytosphingosine in the serum (*p* < 0.05, Fig. 5c), liver (*p* < 0.05, Fig. 5d), and epididymal fat (*p* < 0.05, Supplementary Fig. S9b) increased after *F. plautii* treatment. Notably, the phytosphingosine levels in the liver were much greater than those in other tissues, indicating that phytosphingosine might be enriched in the liver. The effects of *F. plautii* on metabolic disorders were subsequently observed. For the PDC phenotypes, the visible lethargy scores (*p* < 0.05) and greasy fur scores (*p* < 0.01) decreased markedly after *F. plautii* supplementation (Fig. 5e, f). In terms of obesity phenotypes, after 5 weeks of *F. plautii* supplementation, body weight was significantly reduced without changes in food intake (*p* < 0.01, Fig. 5g and Supplementary Fig. S10a), and the amounts of inguinal fat and epididymal fat markedly decreased (*p* < 0.05, Supplementary Fig. S10b). Moreover, H&E staining of adipose tissue revealed a considerable decrease in adipocyte size in epididymal fat (*p* < 0.05, Fig. 5h). In terms of glucose metabolism phenotypes, compared with those in the PBS1 group, fasting serum glucose (*p* < 0.01, Fig. 5i) levels, fasting serum insulin (*p* < 0.001, Supplementary Fig. S10c) levels, and HOMA-IR (*p* < 0.001, Supplementary Fig. S10d) in the *F. plautii* supplementation group were markedly lower. For the uric acid metabolism phenotypes, the level of serum uric acid decreased markedly after *F. plautii* supplementation (*p* < 0.05, Supplementary Fig. S10e). With respect to lipid metabolism phenotypes, a notable reduction in serum TC (*p* < 0.001) and LDLC (*p* < 0.001) levels was observed (Fig. 5j). H&E staining of liver tissue revealed a considerable decrease in liver lipid accumulation following *F. plautii* supplementation (*p* < 0.05, Fig. 5k). Moreover, liver TC and TG markedly decreased in the *F. plautii* group (*p* < 0.01, Supplementary Fig. S10f, g). Additionally, there was no statistically significant change in the liver ALT or AST levels before or after the intervention (Supplementary Fig. S10h, i). Our results suggest that *F. plautii* supplementation can improve metabolic disorders induced by HFD and fecal transplantation in PDC subjects with normal metabolic indices by increasing the content of phytosphingosine in the gut‒hepatic axis.

### Phytosphingosine alleviates the metabolic disorders induced by a HFD and fecal transplantation in PDC subjects with normal metabolic indices

To further investigate the effects of phytosphingosine on metabolic disorders induced by a HFD and fecal transplantation in PDC subjects with normal metabolic indices, PDN-F mice were given phytosphingosine by intragastric gavage daily for five weeks (Fig. 5l). We used PDN-F mice treated with PBS (PBS2 group) as controls and those treated with low doses (25 mg/kg) or high doses (50 mg/kg) of phytosphingosine as the PhyL group and PhyH group, respectively (Fig. 5l). In terms of the PDC phenotypes, the visible lethargy scores (*p* < 0.05) and greasy fur scores (*p* < 0.05) of the PhyH group were markedly lower than those of the PBS1 group (Fig. 5m, n). With respect to obesity phenotypes, we detected a significant reduction in body weight gain in the PhyH (*p* < 0.05) and PhyL (*p* < 0.05) groups (Fig. 5o) without changes in food intake (Supplementary Fig. S11a). Moreover, the amounts of inguinal fat (*p* < 0.01) and epididymal fat (*p* < 0.01) were markedly decreased in the PhyH groups (Supplementary Fig. S11b), and H&E staining demonstrated a considerable decrease in adipocyte size in the epididymal fat of the PhyH (*p* < 0.01) and PhyL (*p* < 0.05) groups (Fig. 5p). Compared with those in the PBS2 group, the fasting serum glucose (*p* < 0.001 for the PhyH group and *p* < 0.05 for the PhyL group; Fig. 5q), fasting serum insulin (*p* < 0.001, Fig. 5r), and HOMA-IR (*p* < 0.001, Supplementary Fig. S11c) levels in the PhyH and PhyL groups were markedly lower. In addition, phytosphingosine improved oral glucose clearance and insulin sensitivity, as revealed by the GTT and ITT results in type 2 diabetic rats (Supplementary Fig. S12a–f). In terms of uric acid metabolism phenotypes, the level of serum uric acid decreased markedly in the PhyH and PhyL groups (*p* < 0.001, Supplementary Fig. S13a). For the lipid metabolism phenotypes, lower serum TC and LDLC and higher serum HDLC were noted in the PhyH (*p* < 0.001) and PhyL (*p* < 0.01) groups (Fig. 5s). Moreover, H&E staining of liver tissue revealed considerably decreased lipid accumulation in the liver tissue of the PhyH and PhyL groups (*p* < 0.05, Fig. 5t), while liver TC (*p* < 0.05 for the PhyH group) and TG (*p* < 0.01 for the PhyH group and *p* < 0.01 for the PhyL group) markedly decreased in the PhyH and PhyL groups (Supplementary Fig. S13b, c), and there was no statistically significant change in liver ALT and AST levels after the intervention (Supplementary Fig. S13d, e). These results indicate that phytosphingosine improves metabolic disorders induced by HFD and fecal transplantation in PDC subjects with normal metabolic indices.

### Phytosphingosine activates PPARα to improve metabolic disorders

**Fig. 6:**
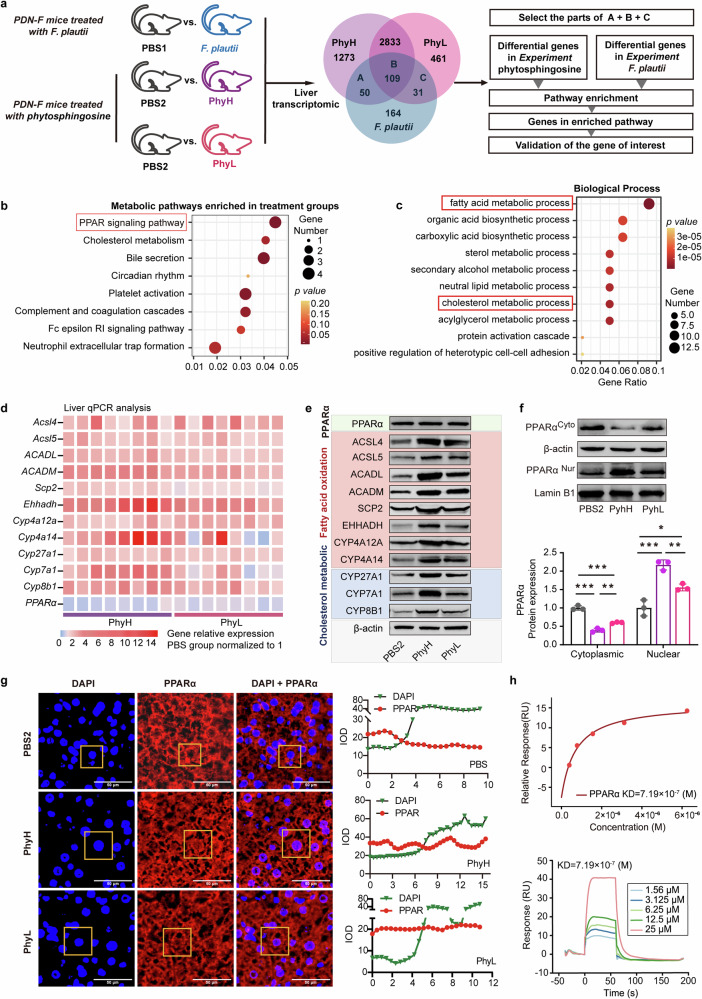
Phytosphingosine activates PPARα to ameliorate metabolic disorders in vivo. **a** Schematic representation of the liver transcriptome analysis. Enrichment analyses of 190 differentially expressed common genes after *F. plautii* intervention or phytosphingosine intervention were performed. **b** Enriched KEGG pathways of the differentially expressed genes. **c** Biological process of a GO functional analysis. **d** Heatmap of hepatic PPARα and downstream gene relative mRNA expression in the PBS2, PhyH, and PhyL groups. The relative gene expression in the PBS group was normalized to 1. Eight biological replicates were performed for each group. **e** Immunoblot analysis of hepatic PPARα and downstream proteins in the PBS2, PhyH, and PhyL groups (biological replicates for each group). **f** Immunoblot analysis and densitometry analysis of nuclear PPARα and cytoplasmic PPARα in the PBS2, PhyH, and PhyL groups (3 biological replicates for each group). **g** Immunofluorescence analysis and densitometry analysis of DAPI and PPARα and a merged image of DAPI and PPARα in the PBS2, PhyH, and PhyL groups; relative luminance curves of DAPI and PPARα in a representative area (from the cytoplasm to the cytoplasm). **h** SPR binding analysis of phytosphingosine to PPARα. PDN-F mice received fecal slurry from the PDN group daily for 14 consecutive days and were fed a HFD for 5 weeks. In the PBS1 group, the PDN-F mice were gavaged with PBS every day in animal experiment 2. In the *F. plautii* group, PDN-F mice were gavaged with 5 × 10^8^ cfu *F. plautii* every day in animal experiment 2. In the PBS2 group, PDN-F mice were gavaged with PNSs every day in animal experiment 3. In the PhyH group, PDN-F mice were gavaged with 50 mg/kg phytosphingosine every day in animal experiment 3. In the PhyL group, PDN-F mice were gavaged with 25 mg/kg phytosphingosine every day in animal experiment 3. IOD, integral optical density; *K*D, equilibrium association constant; DAPI, 4′,6-diamidino-2-phenylindole. Differences among groups were analyzed by one-way ANOVA with Tukey’s post hoc test. *, *p* < 0.05, **, *p* < 0.01, and ***, *p* < 0.001, respectively. The data in the bar plot are presented as the means ± SD.

**Fig. 7:**
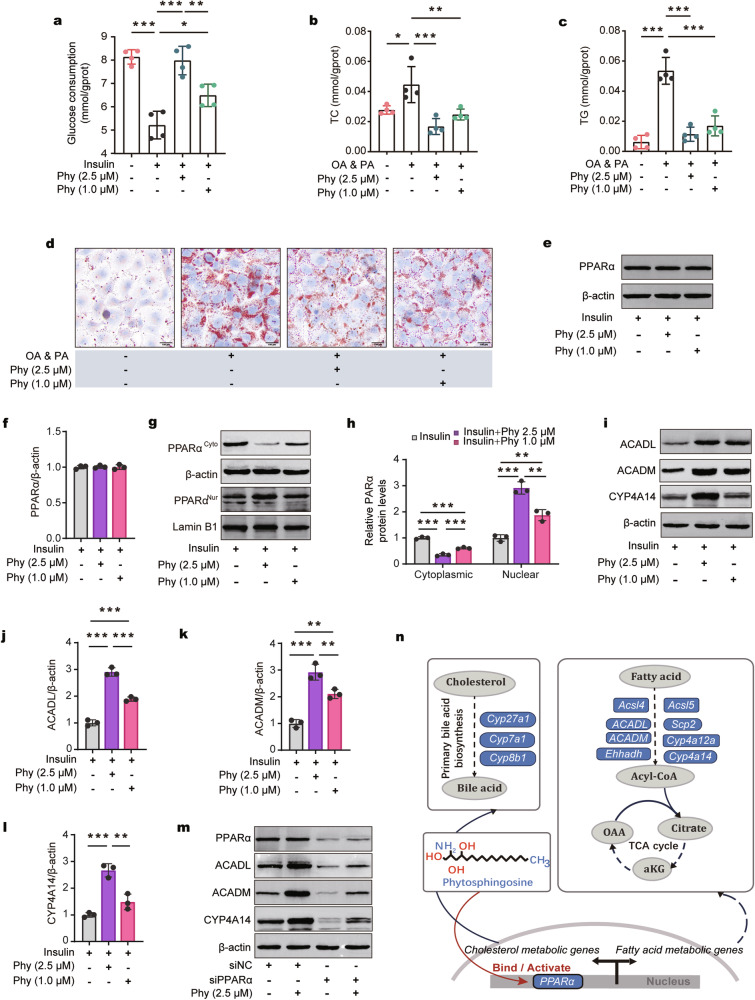
Phytosphingosine activates PPARα to ameliorate metabolic disorders in vitro. **a** Glucose consumption in insulin-induced HepG2 cells treated with phytosphingosine. **b,** **c** Cellular TC (**b**) and cellular TG (**c**) contents in oleic acid-induced HepG2 cells treated with phytosphingosine. *n* = 4 (**a**–**c**). **d** Representative images of Oil Red O staining (scale bar, 100 μm) in OA- and PA-induced HepG2 cells treated with phytosphingosine. **e**, **f** Immunoblot (**e**) and densitometric (**f**) analyses of PPARα in insulin-induced HepG2 cells treated with phytosphingosine. **g**, **h** Immunoblot (**g**) and densitometric (**h**) analyses of nuclear PPARα and cytoplasmic PPARα in insulin-induced HepG2 cells treated with phytosphingosine. **i**–**l** Immunoblot analysis and densitometry analysis of ACADL, ACADM, and CYP4A14 in insulin-induced HepG2 cells treated with phytosphingosine. **m** Immunoblot analysis of PPARα, ACADL, ACADM, and CYP4A14 after PPARα was knocked down in insulin-induced HepG2 cells treated with phytosphingosine. *n* = 3 (**e**–**m**). **n** Schematic representation of the possible mechanism by which phytosphingosine targets PPARα. PA palmitic acid, OA oleic acid, Phy phytosphingosine. Differences among groups were analyzed by one-way ANOVA with Tukey’s post hoc test. *, *p* < 0.05, **, *p* < 0.01, and ***, *p* < 0.001, respectively. The data in the bar plot are presented as the means ± SD.

To elucidate the mechanism by which phytosphingosine improves metabolic disorders, RNA-seq and analysis were performed on liver samples from five groups (the PBS1 vs. *F. plautii* groups; the PBS2 vs. PhyH, or PhyL groups) (Fig. 6a). A total of 55,487 genes were identified. The 190 differentially expressed common genes (DEGs) obtained after *F. plautii* intervention and phytosphingosine intervention (Fig. 6a) were enriched in the PPAR pathway (Fig. 6b). Furthermore, GO enrichment of the DEGs related to the PPAR signaling pathway was performed. The main biological processes were fatty acid metabolic processes and cholesterol metabolic processes (Fig. 6c). Since PPARα is the key regulator of fatty acid metabolism and cholesterol metabolism^32^, we explored the expression of PPARα and its downstream genes after *F. plautii* or phytosphingosine intervention. The results revealed that neither *F. plautii* nor phytosphingosine intervention altered PPARα mRNA or protein levels in the mouse liver (Supplementary Fig. S14a, Fig. 6d; Supplementary Fig. S14b, c, Fig. 6e; Supplementary Fig. S15). However, *F. plautii* and phytosphingosine increased the mRNA levels of 11 genes downstream of PPARα; of these genes, acyl-CoA synthetase long chain family member 4 (*Acsl4*), acyl-CoA synthetase long chain family member 5 (*Ascl5*), acyl-CoA dehydrogenase medium chain (*ACADM*), acyl-CoA dehydrogenase long chain (*ACADL*), sterol carrier protein 2 (*Scp2*), enoyl-CoA hydratase and 3-hydroxyacyl CoA dehydrogenase (*Ehhadh*), cytochrome P450 (Cyp) 4a12a, and *Cyp4a14* are related to fatty acid oxidation, and *Cyp27a1*, *Cyp7a1*, and *Cyp8b1* are related to cholesterol metabolism (Supplementary Fig. S14a and Fig. 6d). Western blot analysis revealed that the changes in the protein levels were consistent with the changes in the mRNA levels of these genes (Supplementary Fig. S14b, c; Fig. 6e; Supplementary Fig. S15). Moreover, after *F. plautii* and phytosphingosine intervention, the levels of glucose metabolism-related proteins (p-IR/IR, p-IRS/IRS, and GLUTs) also increased (Supplementary Figs. S16 and S17). Interestingly, the results of the Western blot analysis of livers (Fig. 6f) or epididymal fat (Supplementary Fig. S18) from the mice revealed that *F. plautii* and phytosphingosine increased the protein nuclear‒cytoplasmic ratio of PPARα. Moreover, the immunofluorescence results in the PhyL and PhyH groups were consistent with the western blot results (Fig. 6g). On the basis of these results, we speculated that there might be molecular binding between phytosphingosine and PPARα. Surface plasmon resonance (SPR) (Fig. 6h) revealed that the *K*D between phytosphingosine and PPARα was 7.19 × 10^−^^7^, and the dual-luciferase reporter system revealed that phytosphingosine can activate the nuclear transcriptional activity of PPARα (Supplementary Fig. S19). To explore the effects of phytosphingosine produced by *F. plautii* on metabolic disorders in vitro, an insulin-resistant human hepatocellular carcinoma (HepG2) cell model and a fatty HepG2 cell model were generated and treated with two doses of phytosphingosine, which were selected based on CCK-8 test results (Supplementary Fig. S20). The results showed that phytosphingosine treatment improved the lipid and glucose metabolic status (Fig. 7a–d). Phytosphingosine intervention did not alter PPARα protein levels in insulin-resistant HepG2 cells (Fig. 7e, f). However, phytosphingosine increased the nuclear-to-cytoplasmic ratio of PPARα (Fig. 7g, h) and resulted in the elevation of key proteins involved in fatty acid oxidation, including ACADM, ACADL, and CYP4A14 (Fig. 7i–l). Furthermore, to determine whether the action of phytosphingosine is PPARα dependent, PPARα-knockdown HepG2 cells were generated. Notably, the effects of phytosphingosine on the protein levels of ACADL, ACADM, and CYP4A14 were attenuated in PPARα-knockdown cells (Fig. 7m and Supplementary Fig. S21). Therefore, both in vivo and in vitro evidence demonstrated that phytosphingosine promoted PPARα nuclear localization and thus activated PPARα-mediated fatty acid metabolism and cholesterol metabolism (Fig. 7n). In addition, SPR (Supplementary Fig. S22) and Western blot analysis (Supplementary Fig. S23) revealed that *F. plautii* and phytosphingosine could also act in epididymal fat and bind other PPAR receptors, such as PPARβ.

## Discussion

**Fig. 8:**
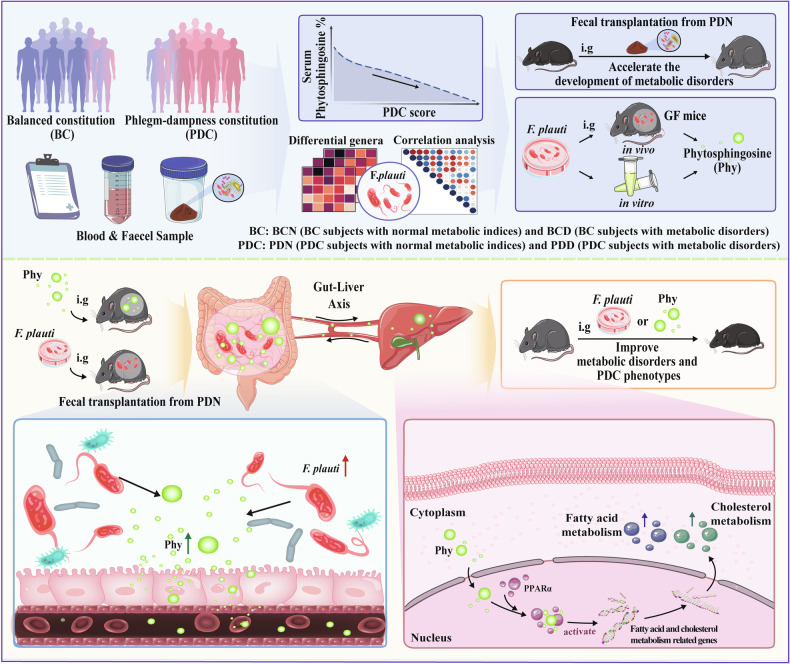
The decrease in *F. plautii* and its product, phytosphingosine, predisposes individuals with phlegm-dampness constitution to metabolic disorders. (**1**) Compared with BC subjects, PDC subjects have distinct gut microbiota and serum metabolite profiles. (**2**) Decreases in *F. plautii* and phytosphingosine were associated with higher PDC scores. (**3**) The bacteria *F. plautii* biosynthesizes phytosphingosine. (**4**) *F. plautii* and serum phytosphingosine levels decreased in PDC subjects with normal metabolic indices. (**5**) Fecal transplantation from PDC subjects with normal metabolic indices accelerated the development of metabolic disorders in mice. (**6**) *F. plautii* or phytosphingosine significantly improved metabolic disorders and PDC phenotypes. (**7**) Phytosphingosine produced by *F. plautii* binds to PPARα and activates the nuclear transcription of PPARα.

PDC is a constitutional type closely related to metabolic disorders in TCM. Without effective intervention, individuals with PDCs are predisposed to develop metabolic disorders^16^. To our knowledge, this study is the first to clarify that gut bacteria and their metabolites contribute to gene expression changes related to metabolic disorders in PDC individuals and increase the predisposition of individuals with PDC to metabolic disorders (Fig. 8). First, through comparative analysis of the gut microbial and serum metabolic characteristics of 167 PDC and 42 BC subjects, we revealed that PDC subjects have distinct gut microbiota and serum metabolite profiles. Second, through correlation analysis, we determined that serum phytosphingosine has the most significant negative correlation with PDC scores. Subsequent experiments demonstrated that *F. plautii* could biosynthesize phytosphingosine, which was negatively correlated with the PDC score. Third, we found that both *F. plautii* and its product, phytosphingosine, decreased in PDC subjects with normal metabolic indices. Fecal transplantation from these individuals accelerated the development of metabolic disorders in mice. However, supplementation with *F. plautii* and phytosphingosine ameliorated metabolic disorders by increasing phytosphingosine levels in the gut‒hepatic axis. Finally, we verified that phytosphingosine can directly bind to hepatic PPARα and activate its nuclear transcription activity, thereby regulating downstream gene expression. These results provide a biological basis for the susceptibility of PDC subjects to metabolic disorders and suggest a new approach to prevent the onset of metabolic disorders even in the preclinical stage.

In this study, we found that PDC scores were significantly positively correlated with metabolic indices. The PDC subjects had a greater degree of metabolic disorders, and the proportion of metabolic diseases in the PDC subjects was greater than that in the BC subjects. These results are consistent with our previous research^20^. However, the biological basis behind this clinical phenomenon needs to be further revealed. TCM recognizes the human body from the perspective of holism and holds that the tissues and organs of the body are not isolated but interrelated^11^. Previous studies have shown that gut microbiota-generated metabolites can be absorbed into the blood circulation and affect host phenotypes, metabolism, and the tissues and organs of the whole body. Thus, the gut microbiota has emerged as a promising avenue for elucidating TCM principles^23^. Moreover, PDC represents a physiological state in which the transport and transformation ability of the spleen and stomach are decreased, and phlegm and dampness accumulate throughout the whole body. The spleen and stomach, as defined by TCM, cover the whole digestive tract in Western medicine and are closely related to the gut microbiota. Therefore, the PDC might be closely related to the gut microbiota.

Previously, our team and others reported that individuals with PDC have a distinctive gut microbial structure^29–31^. In this study, we further revealed that individuals with PDC have distinctive serum metabolite profiles. Furthermore, we found that the gut microbiota structure and serum metabolic profiles of PDC subjects with normal metabolic indices (~7.78% of PDC subjects) were different from those of BC subjects with normal metabolic indices but similar to those of PDC subjects with metabolic disorders. Therefore, we believe that through the TCM diagnostic method of PDC diagnosis, a population with changes in the gut microbiota and related metabolites before metabolic disorders occur could be identified. This might be related to the PDC being diagnosed by collecting PDC phenotypic characteristics via the PDC scoring system rather than relying on metabolic indices^11^. However, the changes that occur in the gut microbiota and metabolites before the occurrence of metabolic disorders have not been described. Using correlation analysis, we highlight the metabolite phytosphingosine, which has the most significant negative correlation with PDC scores, and the bacteria *F. plautii*, which is closely related to it. Interestingly, *F. plautii* and serum phytosphingosine levels are negatively correlated with the degree of metabolic disorders, and both are reduced in PDC subjects with normal metabolic indices. Fecal transplantation from PDC individuals with normal metabolic indices can accelerate metabolic disorders. On the basis of these results, we believe that the gut microbiota and its metabolites could explain the biological basis behind the concept of TCM and link the concept of TCM with modern medicine^22,23,33^. Decreases in *F. plautii* and serum phytosphingosine levels may represent a precursor of metabolic disorders and predispose individuals with PDC to metabolic disorders.

We subsequently explored the relationship between *F. plautii* and phytosphingosine. Phytosphingosine is present in certain tissues of mammals, such as the epidermis and small intestine, but is more common in the plasma membranes of bacteria and fungi. Phytosphingosine is the main sphingosine base in fermented food^34^. In this study, we confirmed that *F. plautii* directs the biosynthesis of phytosphingosine both in vitro and in germ-free mice. *F. plautii* is the main active *Flavonifractor* species, and *Flavonifractor* is a close relative of *Sphingobacteria*^35,36^. A previous study demonstrated that *Sphingobacteria* can produce sphingosine (analogs of phytosphingosine), which supports our results^37^. *F. plautii* is a beneficial bacterium that can protect against increased arterial stiffness^38^. Here, we confirmed that *F. plautii* can biosynthesize phytosphingosine and that it can improve various metabolic disorders.

Next, given the central role of glucose‒lipid metabolism in metabolic disorders, we explored the therapeutic effect and mechanism of phytosphingosine on glucose‒lipid metabolic disorders. Many previous studies have shown that phytosphingosine has antibacterial and protective effects on the skin^39,40^. Some studies have reported that it lowers blood glucose and lipid levels^41,42^, which is consistent with our findings. By interacting with the intestinal tract and adipose tissue, the liver plays a key role in various aspects of glucose‒lipid metabolism. Furthermore, we revealed that phytosphingosine can directly bind to PPARα, activating its nuclear transcription activity in the liver or epididymal fat. PPARα is a transcription factor that belongs to the nuclear receptor superfamily. As a gene regulator of metabolic pathways, PPARα is a key player in regulating energy and lipid metabolism, glucose homeostasis, adipogenesis, and inflammation^43–45^. This may be an important mechanism by which DEGs from PDC individuals are enriched in the PPAR signaling pathway and are associated with metabolic disorders, low immunity and an inflammatory phenotype^21,46^. Here, we verified that phytosphingosine activated the nuclear transcription of PPARα and improved glucose‒lipid metabolism. Moreover, *F. plautii* and phytosphingosine could also act on other PPAR receptors. In the future, the detailed mechanism needs to be explored.

Regarding to phytosphingosine, it is inevitable to think of sphingosine. Sphingosine and phytosphingosine are sphingoid bases and are both components of sphingolipids, but they have different physiological and pharmacological activities. Excess sphingosine is widely known to be a toxicant since it induces apoptosis and binds long-chain fatty acids to produce ceramides, which can cause insulin resistance, lipid accumulation, inflammation, and apoptosis^47,48^. However, recent studies have shown that glycosylated or homoserine sphingosine and ceramides produced by the gut microbiota can regulate host sphingosine and ceramide metabolism and ameliorate metabolic disorders^49,50^. Glycosylated or homoserine modifies the structure of sphingosine and ceramides, whereas phytosphingosine and sphingosine differ in the structure of the mother nucleus. These findings suggest that phytosphingosine may have a positive effect on sphingosine and ceramide metabolism, which will also be the direction of our future exploration.

In modern medicine, identifying changes in precursors before the occurrence of diseases is important, as preventing and blocking disease processes is beneficial. For example, systems biomedicine, a relatively new discipline that combines experimental observations with computer modeling to simulate disease, has been gradually applied to identify precursor changes before diseases occur^51,52^. Claudio Franceschi from the University of Bologna (Bologna, Italy) established a model by collecting data related to the gut microbiota, inflammation and aging and reported that before the onset of diabetes, the gut microbiota exhibited changes related to the inflammatory response^10^. In this study, based on the cross-sectional clinical investigations of PDC subjects and experimental validation, we revealed that the decrease in *F. plautii* and its product, phytosphingosine, can contribute to metabolic disorders, which may be a precursor of metabolic diseases. Through the diagnosis of PDC, people with these precursor changes can be identified. These findings indicate that TCM diagnosis is a method that can aid in the early detection and prevention of diseases. In addition, this study confirmed that the phenotypic characteristics of PDC have a biological basis, and similar research on other constitutional types can also be performed in the future to contribute to disease prevention.

This research has several limitations that should be noted. Owing to the age distribution of PDC subjects, it is challenging to recruit matched subjects for clinical research. Moreover, owing to restrictions related to clinical specimen collection, only blood and fecal samples were collected from our cohort. The availability of tissues closely related to metabolism, such as the liver, intestine, and adipose tissue, would strengthen our conclusions. Finally, in the mouse fecal transplantation experiment, we assessed the transfer of the PDC phenotype. Owing to the limited capacity of rodents to mimic humans, we were only able to observe visible lethargy and greasy fur statuses in mice; we were unable to observe symptoms such as upper eyelid edema and thick tongue coatings in the mouse model. Future studies to address these key issues may help gain a better understanding of the pathogenesis of metabolic disorders via TCM.

## Materials and methods

### Key materials and software

The key materials and software were showed in Table 1.

**Table 1:** Key materials and software.

Reagent or resource	Source	Identifier
Antibodies		
Primary antibody against PPARα	Abcam Co. Ltd., CA, USA	ab126285
Primary antibody against ACADM	Proteintech Group, Inc., Wuhan, China	55210-1-AP
Primary antibody against ACADL	Proteintech Group, Inc., Wuhan, China	17526-1-AP
Primary antibody against CYP4A14	Abcam Co. Ltd., CA, USA	ab140635
Primary antibody against CYP7A1	Santa Cruz Biotechnology Inc., CA, USA	sc-518007
Primary antibody against CYP8B1	Abcam Co. Ltd., CA, USA	ab236607
Primary antibody against CYP27A1	Proteintech Group, Inc., Wuhan, China	14739-1-AP
Primary antibody against ACSL4	Proteintech Group, Inc., Wuhan, China	22401-1-AP
Primary antibody against ACSL5	Proteintech Group, Inc., Wuhan, China	15708-1-AP
Primary antibody against SCP2	Abcam Co. Ltd., CA, USA	ab140126
Primary antibody against EHHADH	Proteintech Group, Inc., Wuhan, China	26570-1-AP
Primary antibody against β-actin	Proteintech Group, Inc., Wuhan, China	81115-1-RR
Goat secondary antibody against rabbit	Proteintech Group, Inc., Wuhan, China	RGAR001
Goat secondary antibody against mouse	Proteintech Group, Inc., Wuhan, China	RGAM001
Primary antibody against GLUT2	Abcam, Ltd., Shanghai, China	ab152680
Primary antibody against GLUT4	Abcam, Ltd., Shanghai, China	ab33780
Primary antibody against p-IR-β	Cell Signaling Technology, Inc., Shanghai, China	#2381
Primary antibody against p-IRS	Cell Signaling Technology, Inc., Shanghai, China	#3026
Primary antibody against IR-β	Cell Signaling Technology, Inc., Shanghai, China	#3025
Primary antibody against IRS	Cell Signaling Technology, Inc., Shanghai, China	#2382
Recombinant protein and chip		
Human PPARα Protein	Sino Biological Inc., Beijing, China	12080-H07E
Human PPARβ/δ Protein	Cloud-clone corp., Wuhan, China	RPC041Hu01
Human PPARγ Protein	Sino Biological Inc., Beijing, China	12019-H20B
Insulin	Beijing Solarbio Science & Technology Co., Ltd., Beijing, China	I8830
CM5 sensor chip	GE Healthcare, Chicago, IL, USA	BR100012
Experimental models: cell lines		
HepG2 (human hepatocellular carcinoma) cells	Procell Life Science&Technology Co., Ltd., Wuhan, China	CL-0103
C57BL/6J mouse	Charles River Biotechnology Co., Ltd, China	213
Wistar rat	Charles River Biotechnology Co., Ltd, China	102
GF C57BL/6J mouse	Gempharmatech Co., Ltd	750
Critical commercial assays		
Dual Luciferase Reporter Gene Assay kits	Hanbio Biotechnology Co. Ltd., Shanghai, China	20231017GX-DLR01
PPARα pcDNA3.1 plasmid	Hanbio Biotechnology Co. Ltd., Shanghai, China	20231017GX-PC04
PPRE pGL4.26 plasmid	Hanbio Biotechnology Co. Ltd., Shanghai, China	20231017GX-PC03
pRL-TK plasmid	Hanbio Biotechnology Co. Ltd., Shanghai, China	20231017GX-PC06
Rat rxra pcDNA3.1-3xFlag-T2A-EGFP	Hanbio Biotechnology Co. Ltd., Shanghai, China	20231017GX-PC02
Sodium palmitate/sodium oleate kit	Kunchuang Biotechnology, Xian, China	2
PPARα siRNA	Hanbio Biotechnology Co. Ltd., Shanghai, China	20230809GX-SI02
Control siRNA	Hanbio Biotechnology Co. Ltd., Shanghai, China	20230809GX-SI02
Lipo 8000 transfection reagent	Beyotime Biotechnology, Shanghai, China	C0533
Insulin ELISA Kit for	Cloud-clone corp., Wuhan, China	CEA448Mu
Opti-MEM	Invitrogen Co., Carlsbad, CA, USA	31985070
Oil Red O Stain Kit	Solarbio Co. Ltd., Beijing, China	G1262
Mouse GPT/ALT kit	Grace Biotechnology Co. Ltd., Suzhou, China	G0423W
Mouse GOT/AST kit	Grace Biotechnology Co. Ltd., Suzhou, China	G0424W
Mouse TG Content Assay Kit	Grace Biotechnology Co. Ltd., Suzhou, China	G0910W
Mouse TC Content Assay Kit	Grace Biotechnology Co. Ltd., Suzhou, China	G0909W
Taq Pro Universal SYBR qPCR master mix	Vazyme Biotechnology Co. Ltd., Nanjing, China	Q712-02
E.Z.N.A.â Soil DNA kit	Omega Bio-Tek Co. Ltd., Norcross, GA, USA	D5625-02
HE Staining Kit	Solarbio Co. Ltd., Beijing, China	G1120
HiScript^®^ III All-in-one RT SuperMix Perfect for qPCR	Vazyme Biotechnology Co. Ltd., Nanjing, China	R333
Software and algorithms		
BiacoreTM Insight Software	Cytiva	https://www.cytivalifesciences.com/en/us
ImageJ	National Institutes of Health (NIH)	https://imagej.nih.gov/ij/
Adobe Illustrator	Adobe	https://www.adobe.com/products/illustrator.html
SPSS 16.0	IBM SPSS	https://www.ibm.com/hk-en/products/spss-statistics
*R* studio	RStudio	https://www.r-project.org/
Graphpad Prism 7	GraphPad	https://www.graphpad.com/scientificsoftware/prism/
Bacterial and Chemicals		
*Flavonifractor plautii*	Guangdong Microbial Culture Collection Center (GDMCC)	GDMCC NO.1.1268
Phytosphingosine (≥ 98%)	Aladdin	D136389 CAS: 554-62-1
neomycin sulfate (600 μg/mg)	Macklin	N814740 CAS: 1405-10-3
metronidazole (≥ 98%)	Macklin	M813526 CAS: 443-48-1
Ampicillin (≥ 98%)	Macklin	A830931 CAS: 69-53-4
Vancomycin (≥ 950 μg/mg)	Macklin	V871983 CAS: 1404-90-6
Recombinant Human Insulin	Solarbio Co. Ltd., Beijing, China	I8830
TRIzol	Vazyme Biotechnology Co. Ltd., Nanjing, China	#R411
Streptozotocin	Sigma‒Aldrich, Inc., Shanghai, China	S0130
Other		
Automatic Biochemistry Analyzer	Beckman Coulter, Inc. CA, USA	AU480
Glucometer	Bayer, Leverkusen, Germany	D2ASCCONKIT
Normal chow diet	SiPeiFu (Beijing) Biotechnology Co., Ltd, China	SPF-F02-003
High fat Diet	SiPeiFu (Beijing) Biotechnology Co., Ltd, China	010139
High fat Diet With 60 kcal% diet	SYSE BIO Co., Ltd, Changzhou, China	PD6001

### Volunteer recruitment and data collection

The Institutional Review Board—Ethics Committee and the Committee for Beijing University of Chinese Medicine approved the protocols of the human study (2017BZHYLL0502). The research performed in this study complied with all relevant ethical regulations. All experiments adhered to the Declaration of Helsinki. Written informed consent was obtained from all participant. The inclusion criteria were as follows: 1. met the criteria for determining the BC/PDC constitution (ZYXH/T157-2009), 2. aged between 18 and 50 years, and 3. had at least 1 year of living in Beijing. The exclusion criteria were as follows: 1. the use of antibiotics, gastrointestinal stimulants, microecological regulators, or endocrine-affecting drugs in the past three months; 2. weight loss by any pharmacological means in the past three months; 3. a history of gastrointestinal surgery; 4. the presence of a serious illness, infectious disease or mental disorder; or 5. alcoholism.

According to the industry standard (version: ZYYXH/T157-2009; Supplementary Method Section 1), individuals were diagnosed with PDC when the PDC score was ≥ 40, and individuals were diagnosed with BC when the BC score was ≥ 60 and the unbalanced constitution score was < 30. A total of 209 subjects, including 167 PDC subjects and 42 BC subjects, were enrolled. All the clinical characteristics are shown in Supplementary Table S1. The PDC subjects were further divided into two groups according to the diagnostic criteria for metabolic disorders (Supplementary Method Section 2): the PDN group (*n* = 13, PDC subjects with normal metabolic indices) and the PDD group (*n* = 154, PDC subjects with metabolic disorders). Similarly, the PDC subjects were also further divided into two groups according to the diagnostic criteria for metabolic disorders: the BCN (*n* = 36, BC subjects with normal metabolic indices) group and the BCD (*n* = 6, BC subjects with metabolic disorders) group.

The waist circumference was measured by circling the abdomen in a horizontal direction 5 cm above the umbilicus. The hip circumference was measured at the most prominent part of the buttocks. Blood pressure was measured with a standard mercury sphygmomanometer by specially trained nurses when the subjects were in a seated position after ≥ 5 min of rest. Blood and stool samples were collected from the participants after an overnight fast. The peripheral blood samples were incubated at room temperature for at least 30 min and centrifuged at 3000× *g* for 5 min, after which the supernatant was purified and stored at −80 °C. The subjects were given a stool sampler and provided detailed instructions for sample collection. Immediately after collection, the fresh stool samples were transported to the laboratory and frozen at −80 °C.

In this cohort, we characterized the gut microbiome and serum metabolome and identified microbial and metabolic characteristics (Fig. 1a). The collected metadata covered demographic characteristics, health status, disease history and drug treatment, as well as the TCM constitution scale.

### Untargeted metabolomics analysis

The samples were thawed on ice, and QC samples were also prepared by mixing and blending equal amounts of each sample. LC–MS/MS analyses were performed via an ultra-performance liquid chromatography (UPLC) system (Thermo Fisher Scientific, USA) with a UPLC BEH C18 column (1.7 μm, 2.1 mm × 100 mm, Waters, USA) coupled to a Q Exactive HF-X mass spectrometer (Thermo Fisher Scientific, USA) in both positive and negative ionization modes. The original LC‒MS/MS data were processed via Progenesis O1 V2.3 software (Nonlinear Dynamics, Newcastle, UK) for baseline filtering, peak identification, integral and retention time correction, peak alignment, and normalization. The extracted data were then processed to generate a data matrix consisting of a combination of the data obtained in the positive and negative ion modes. OPLS–DA was applied to discriminate the different groups. The metabolites that differed between PDC subjects and BC subjects were identified on the basis of the following criteria: VIP > 1.0, two-tailed Student’s *t*-test (*p* < 0.05), and fold change (< 0.8 or > 1.2). Metabolic pathway enrichment analysis of the differentially abundant metabolites was performed via the Kyoto Encyclopedia of Genes and Genomes (KEGG) database.

### DNA extraction and 16S rRNA gene V3-V4 region sequencing

Following the instructions provided with the EZNA^®^ soil kit, total DNA was extracted from the stool samples. A NanoDrop2000 (Thermo Fisher Scientific, USA) was used to detect and calculate the DNA purity and concentration, and a 1% agarose gel was used to determine the quality of the extracted DNA. The 343 F (5′-TACGGRAGGCAGCAG-3′) and 798 R (5′-AGGGTATCTAATCCT-3′) primers were used for PCR amplification of the V3–V4 hypervariable region of the DNA samples. The amplicon quality was visualized by gel electrophoresis, and the amplicon was purified with AMPure XP beads and amplified through another round of PCR. After another round of purification with AMPure XP beads, the final amplicon was quantified via a Qubit dsDNA assay kit. Equal amounts of purified amplicon were pooled for subsequent sequencing.

### Sequencing data analysis

Paired-end reads were generated and assigned to each sample on the basis of their barcodes and then merged via Flash (version 1.2.11) software. Through high-quality filtering, reads with ambiguous or homologous sequences or less than 200 bp were removed from the raw tags to acquire clean tags via the split_libraries (version 1.8.0) function of QIIME software (version 1.8.0). The downstream bioinformatic analyses were performed via EasyAmplicon v1.0^53^. We then discarded low-abundance sequences (*n* < 8) via the derep_fullength command in VSEARCH software (v2.15). The nonredundant sequences were denoised into amplicon sequence variants (ASVs) via the -unoise3 command in USEARCH software (v10.0). The feature (ASV) table was created with Vsearch–usearch_global. We analyzed the high-quality reads via USEARCH to remove chimeric and organelle sequences, which resulted in the generation of 3076 ASVs. The representative sequences for each ASV were classified, and the Ribosomal Database Project (RDP) classifier (version 11.5) was then used to assign taxonomic data to each sequence. The sequences of all the samples were rarefied to 30000 for downstream diversity analysis. The α diversity was assessed via species richness indices and Shannon diversity indices. The β diversity was calculated via PCoA. Random forest and AUC calculations were performed on the oebiotech platform (https://cloud.oebiotech.cn/task/), a free online data analysis website.

### Metagenomic sequencing and analysis

For shotgun sequencing, Illumina libraries were prepared via a Nextera DNA Sample Prep Kit (Illumina) according to the manufacturer’s protocol and sequenced via the Illumina NovaSeq platform to generate 2 × 150-bp paired-end reads. The Illumina sequence data and low-quality reads were filtered and trimmed via Trimmomatic version 0.36 with the default parameters. After valid reads were obtained, metagenome assembly was performed via SOAPdenovo2 (v2.04). Gaps inside the scaffold were used as breakpoints to break the scaffold into new contigs (ScafContig), and new contigs with a length of 200 bp were retained. The ORFs of the assembled scaffolds were predicted via Prodigal (v2.6.3), and these scaffolds were translated into amino acid sequences. Nonredundant gene sets were built for all the predicted genes via CDHIT (v4.6.7). The clustering parameters were 95% identity and 90% coverage. The longest gene was selected as a representative sequence of each gene set. The clean reads of each sample were aligned against the nonredundant gene set (95% identity) via Bowtie2 (v2.2.9), and the abundance information of the gene in the corresponding sample was obtained. The taxonomy of the species was obtained from the corresponding taxonomy database of the NR Library, and the species abundance was calculated from the corresponding abundance of the genes. To construct the abundance profile for the corresponding taxonomy level, statistical analyses of the abundance data were performed at the Domain, Kingdom, Phylum, Class, Order, Family, Genus, and Species levels. PCoA and plotting of the abundance spectrum of the species or functional abundance spectrum were conducted via R software, and the results from the equidistant PCoA were calculated and analyzed.

### Preparation and in vitro culture of *F. plautii*

*F. plautii* was obtained from the Guangdong Microbial Culture Collection Center (GDMCC). All the samples were processed and cultured under anaerobic conditions in an ELECTROTEK AW 400SG workstation at 37 °C. The culture media, PBS and all other materials that were used for culturing were placed in the anaerobic cabinet 24 h before use to allow their acclimation to anaerobic conditions. *F. plautii* lyophilized powder was resuscitated on blood agar plates and then incubated in modified BHI broth.

For the in vitro experiment, the cultured *F. plautii* were diluted 1:500, inoculated in BHI broth and cultured for 24 h. *F. plautii* cultures were collected after 0, 2, 6, and 10 h for the phytosphingosine-targeting assay.

In vivo, the bacterial cells were pelleted by centrifugation at 3000× *g* and 4 °C for 5 min. The cells were then washed and resuspended in sterile reduced PBS to obtain a density of 5 × 10^8^ colony-forming units (CFUs) per 200 μL. The bacterial suspension was freshly prepared for the animal experiments.

### Measurement of the phytosphingosine level

The level of phytosphingosine was analyzed via ultra-performance liquid chromatography‒tandem mass spectrometry (UPLC‒MS/MS). Briefly, the samples were extracted with extraction solution (chloroform:methanol = 9:1). After 5 min of vortexing and 10 min of centrifugation at 12,000× *g*, 400 μL of the supernatant was concentrated to complete dryness at 20 °C and then reconstituted with 100 μL of methanol. Phytosphingosine was separated on an ACQUITY HSS T3 column (i.e., 2.1 × 100 mm, 1.8 μm) using water and methanol as the mobile phases and detected via MRM on a QTRAP 6500 mass spectrometer (Sciex, Concord, Canada). Quantification was performed via internal and external standard curves.

### Mouse experiments

C57BL/6J mice were maintained at a temperature of 30 ± 2 °C with 45%–70% humidity under a 12-h light/dark cycle (light from 7 a.m. to 7 p.m.) and standard specific-pathogen-free (SPF) or GF conditions. SPF mice were purchased from Beijing Vital River Laboratory Animal Technology Co., Ltd. All the mice were males, aged 7 weeks and weight matched at the start of the experiments. The mice were fasted for 8 h before being killed. The weekly food consumption was measured per cage. All the mice were fed a HFD (58.6% basal feed, 15% lard, 20% sucrose sugar, 5% casein, 1.2% cholesterol, and 0.2% sodium cholate) (SiPeiFu (Beijing) Biotechnology Co., Ltd., China) during the intervention period.

#### Animal experiment 1. Fecal transplantation experiment

Five subjects from the BCN group and five from the PDN group who were matched with respect to age, sex and BMI were selected for the fecal transplantation experiment (Supplementary Table S2). Five hundred milligrams of fresh mixed stools obtained from the PDN group or BCN group were suspended in 5 mL of PBS buffer. The stool samples were pooled to obtain the fecal slurry. All the mice were treated with a previously described antibiotic cocktail (vancomycin, 100 mg/kg; neomycin sulfate, metronidazole, and ampicillin, 200 mg/kg) over two cycles of treatment (5 days of treatment and one day of suspension)^54^. Following antibiotic treatment, the recipient mice received fecal slurry from the PDN group or BCN group daily for 14 consecutive days and were fed a HFD for 5 weeks. Blood, fecal, liver, and adipose tissue samples were harvested at the end of the experiment. The experiment was approved by the Medical Ethics Committee, Shandong University of Technology (YLX20220615).

#### Animal experiment 2. Treatment of a humanized animal model of PDC with F. plautii

Antibiotic-treated mice received a fecal slurry from the PDN group and were fed a HFD to establish the mouse model described above. The bacterial suspension containing *F. plautii* was gavaged into the model mice at a dose of 5 × 10^8^ cfu for 5 weeks (*F. plautii* group). Sterile PBS was used as a control (the PBS1 group). Blood, fecal, liver, and adipose tissue samples were harvested at the end of the experiment. The experiment was approved by the Medical Ethics Committee, Shandong University of Technology (YLX20220906).

#### Animal experiment 3. Treatment of a humanized animal model of PDC with phytosphingosine

Phytosphingosine (D136389) was obtained from Aladdin and mixed with sterile PBS to form a suspension. Antibiotic-treated mice receiving the fecal slurry from the PDN group were gavaged with PBS as the PBS2 group and gavaged with 25 or 50 mg/kg phytosphingosine as the PhyL group or PhyH group on a daily basis for 5 weeks. The mice were fasted for 8 h before being killed. Blood, liver, and adipose tissue samples were harvested at the end of the experiment. The experiment was approved by the Medical Ethics Committee, Shandong University of Technology (YLX20220905).

#### Animal experiment in which F. plautii colonized germ-free mice

Male germ-free C57BL/6J mice were obtained from GemPharmatech Co., Ltd. The germ-free mice (8 weeks old) fed a HFD were randomly divided into two groups: the PBS-G group (the germ-free mice were gavaged with PBS every day) or the *F. plautii-*G group (the germ-free mice were gavaged with 5 × 10^8^ cfu *F. plautii* every day). After 10 days of administration, blood, fecal, liver, and adipose tissue samples were harvested. The colonization of germ-free C57BL/6J mice with the *F. plautii* strain was conducted with the approval of the Institutional Animal Care and Use Committee of Gempharmatech Co., Ltd. (AP#: GPTAP20230925-4).

In both batches of experiments, no mice died during the experiment, and body weight and food intake were measured once a week. During the experiment, animal pain was alleviated using isoflurane anesthesia. After the experiment, the animals were euthanized with carbon dioxide.

### Body condition scoring

For PDC phenotype scoring, the mice were scored on a scale of 0–3 in two categories as follows: (1) visible lethargy: 3 points if stationary unless stimulated, 2 points if showing mildly decreased movement, 2 points if showing moderately decreased movement, 0 points if normal; (2) greasy fur: 3 points if severely greasy with poor grooming, 2 points if mildly greasy, 1 point if moderately greasy, 0 points if normal^55^. Visible lethargy and greasy fur are analogous to heavy body and greasy skin in humans, respectively.

### Biochemical analyses

Fasting blood samples were centrifuged at 4000× *g* for 5 min at 4 °C to obtain the serum. The main tissues were immediately excised and stored in liquid nitrogen for biochemical analyses. The levels of TC, TG, LDLC, HDLC, serum glucose, serum insulin, and uric acid in the serum were measured via a Beckman-Coulter AU480 automated analyzer. The concentrations of TC, TG, AST, and ALT in the liver were determined via commercial kits (Grace Biotechnology Co. Ltd., Suzhou, China) according to the manufacturer’s instructions.

### Histological analyses

For histological analyses, tissue samples were fixed in 4% paraformaldehyde solution for 24 h and then embedded in paraffin. The liver and adipose sections (thickness of ~3 μm) were stained with hematoxylin‒eosin, and the stained samples were observed at ×400 magnification. The liver and adipose lipid levels (% staining area) were analyzed via ImageJ software (https://imagej.nih.gov/ij/). For Oil Red O staining, the fixed HepG2 cells were washed with PBS and stained with Oil Red O working solution according to the kit’s instructions. Briefly, Oil Red O working solution was freshly prepared, and HepG2 cells were dyed for 30 min. Then, the cells were washed with 60% isopropyl alcohol to remove the excess dye and washed with PBS. Mayer hematoxylin was applied for 2 min to further dye the nuclei, which were subsequently washed with PBS. Finally, the stained HepG2 cells were observed with an Olympus microscope (Olympus, Tokyo, Japan) and analyzed via ImageJ software.

### Immunofluorescence

For immunofluorescence analysis, the deparaffinized slides were submerged in citric acid antigen retrieval solution (Solarbio) under steam treatment for 20 min. The tissues were allowed to cool at room temperature for 30 min, subjected to three 3-min washes with PBS, and then permeabilized by incubation with 0.1% Triton-X 100 in PBS for 15 min. The sections were then blocked by incubation with 5% bovine serum albumin in PBS for 30 min at 37 °C, stained with anti-pparα antibody overnight at 4 °C in a humidification chamber, subjected to three 3-min washes with PBS, and then incubated with a fluorescein isothiocyanate-labeled secondary antibody in PBS with 1% BSA at a 1:200 dilution. The unbound secondary antibody on the sections was removed by two washes with PBS, and the tissues were then stained with DAPI for 5 min and mounted with Glycerol Jelly Mounting Medium (Solarbio). The fluorescent signals were viewed under a panoramic scanner (3DHISTECH P250 FLASH).

### Oral glucose tolerance test and insulin tolerance test

Sprague-Dawley (SD) rats were maintained at a temperature of 20 ± 2 °C with 45%–70% humidity under a 12-h light/dark cycle (light from 7 a.m. to 7 p.m.) and standard SPF conditions. All the rats were males, aged 8 weeks and weight matched (180–200 g) at the start of the experiments. The type 2 diabetic rat model was established using streptozotocin and a HFD. The HFD consisted of 36.4% starch, 25.6% butter, 20% protein, 1% cholesterol, and 0.1% bile acid. The mice were injected intraperitoneally with 45 mg/kg STZ, and subsequent oral GTTs were performed on the 7th and 21st days after injection. A total of 16 rats met the standard of type 2 diabetes (FBG ≥ 7.0 mM and 2 h-PG ≥ 11.1 mM) in both GTTs. In total, 16 matched diabetic rats were selected for phytosphingosine prevention. The 8 diabetic rats fed a HFD were grouped as the DM-CON group, and another 8 diabetic rats fed a HFD were administered 50 mg/kg/d phytosphingosine by gavage for 3 weeks as the DM-PHY group (*n* = 8 for all groups). After phytosphingosine prevention, GTTs and ITTs were performed. For the GTTs, oral glucose (2.5 g/kg) was given to 4-h-fasted individually housed rats. All GTTs began at 1 pm, and glucose was measured in whole blood collected from the tail vein using a glucometer at 0, 15, 30, 60, 90, and 120 min after glucose gavage. For ITTs, intraperitoneal insulin (0.5 U/kg) was given to 4-h fasted individually housed mice. All ITTs began at 1 pm, and glucose was measured in whole blood that was collected from the tail vein using a glucometer at 0, 30, 60, 90, and 120 min after insulin injection.

No rats died during the experiment. During the experiment, animal pain was alleviated using isoflurane anesthesia. After the experiment, the animals were euthanized with carbon dioxide.

### Cell culture and treatment

HepG2 cells were purchased from Procell Life Science & Technology Co., Ltd. HepG2 cells were cultured in Dulbecco’s modified Eagle’s medium (DMEM) supplemented with 10% fetal bovine serum and 1% penicillin/streptomycin at 37 °C in a 5% CO_2_ atmosphere. HepG2 cells were used for insulin-resistant model or lipid-loaded model construction and phytosphingosine administration. For the insulin-resistant model, HepG2 cells were incubated with 1 μM insulin for 48 h and then changed to nonserum culture medium for another 12 h. Finally, HepG2 cells were given 1 μM insulin culture medium with or without 1 or 2.5 μM phytosphingosine in DMSO for 48 h. For the lipid-loaded model, HepG2 cells were treated with 500 μM palmitic acid or 1000 μM oleic acid for 48 h, after which 1 or 2.5 μM phytosphingosine was administered for 48 h.

### Liver RNA extraction, sequencing, and processing

Total RNA was extracted from fresh frozen liver tissues via TRIzol reagent. The quantity and quality of total RNA were determined via a NanoDrop 2000 ultramicro-spectrophotometer. Poly(A)-containing mRNA was then enriched via magnetic beads with oligo (dT) and fragmented into small pieces via divalent cations at elevated temperatures. The cleaved RNA fragments were reverse transcribed with Oligo T primers to produce cDNA, and dsDNA samples were generated via DNA polymerase, RNase H enzyme, and T4 DNA ligase. These cDNA fragments were then subjected to end repair, the addition of a single ‘A’ base, and ligation with adapters. The resulting products were purified and amplified via PCR to create the library for sequencing. The Illumina HiSeq X Ten/NovaSeq 6000 platform was used for RNA sequencing. The raw RNA-seq data were trimmed via Prinseq (v0.20.4), and the read quality was assessed via FastQC (v0.11.2). The trimmed reads were aligned to the *Rattus norvegicus* reference genome (release 6) or the *Mus musculus* reference genome (release 10) from the UCSC genome browser database via STAR aligner (v2.7.9a). The number of reads per gene was quantified via HTSeq (v0.9.1). The DEGs between groups were identified via the R package DESeq2 (v1.32.0). KEGG pathway enrichment analyses via the GOseq R package (v1.44.0) were performed after annotation of each gene to the corresponding KEGG orthology based on the KEGGREST package (v1.32.0).

### DNA isolation and quantitative PCR

Following the instructions provided with the E.Z.N.A.® Soil DNA Midi Kit (Omega Bio-Tek Co. Ltd., Norcross, GA, USA), total DNA was extracted from the stool samples. Briefly, 1 μL of gDNA was added to 7.5 μL of 2 × Taq Pro Universal SYBR qPCR Master Mix (Vazyme-Q712) with 0.7 μL of each forward and reverse primer pair, and molecular biology-grade H_2_O was then added to obtain a final volume of 20 μL. qPCR detection was performed with the Step One Plus real-time PCR system and software (Applied Biosystems, Foster City, CA, USA) under the following conditions: 95 °C for 5 min followed by 40 cycles of 95 °C for 20 s, annealing at 55 °C for 20 s and extension at 72 °C for 40 s. The computed CT value was substituted into the standard curve formula *y* = −3.6206x + 31.549 as previously described^56^, and the logarithm of gene copy number *x* was calculated. The sequences of the primers used in this study are shown in Supplementary Table S4.

### RNA extraction and RT‒qPCR

Total RNA was extracted from the liver tissue of the animals via TRIzol reagent (Vazyme, Nanjing, China) according to the manufacturer’s instructions. cDNA was generated via reverse transcription of 1–2 µg of total RNA with a reverse transcription enzyme (Vazyme, Nanjing, China). The results from the gene expression analysis are presented as percentages of the expression of each gene normalized to that of glyceraldehyde-3-phosphate dehydrogenase (GAPDH) as a reference. qPCR detection was performed with the Step One Plus real-time PCR system and software (Applied Biosystems, Foster City, CA, USA). Briefly, 2 μL of cDNA was added to 10 μL of 2× Taq Pro Universal SYBR qPCR Master Mix (Vazyme-Q712) with 0.4 μL of each forward and reverse primer pair, and molecular biology-grade H_2_O was added to reach a final volume of 20 μL. qPCR was performed via the LightCycler 480 System (Roche Diagnostics International AG, Switzerland) with the following conditions: 95 °C for 30 s followed by 40 cycles of 95 °C for 10 s and annealing/extension at 60 °C for 30 s. The relative expression levels were calculated on the basis of the expression of GAPDH via the 2^-ΔΔCT^ method. The sequences of the primers used in this study are shown in Supplementary Table S4.

### Western blot

Total protein from mouse liver samples or cells was extracted with RIPA buffer and quantified with a BCA protein assay kit. After the proteins were resolved via SDS‒PAGE, they were transferred to PVDF membranes, blocked with 5% bovine serum albumin at room temperature for 1 h, and incubated with specific primary antibodies at 4 °C overnight. The membranes were incubated with the corresponding secondary antibodies. Densitometric analysis was then performed with ImageJ software.

### Transient dual-luciferase reporter assay

Briefly, a dual-luciferase reporter assay was performed via the transfection of a plasmid with PPRE sequences and internal control vectors (PGL6 promoter) into HEK-293 cells. Luciferase activity was detected via a dual luciferase reporter assay system (Beyotime) with an i3X spectrometer (Molecular Device SpectraMax). The expression level is expressed as the ratio of firefly luciferase activity to Renilla luciferase activity.

### Surface plasmon resonance

Interactions between PPARα and phytosphingosine were analyzed via the Biacore T200 system at 25 °C. Recombinant PPARs (LBDs) were immobilized on a CM5 sensor chip. The level of immobilized PPARs (LBD) was ~14000 RU. Phytosphingosine was injected at various concentrations as an analyte, and 5% DMSO in PBS-P (5% DMSO + PBS + 0.05% P20) was used as running buffer. To explore whether phytosphingosine had any effect on the interactions with PPARs, phytosphingosine at 1.56 μM, 3.125 μM, 6.25 μM, 12.5 μM, or 25 μM was flowed through the chip with a contact time of 60 s and a dissociation time of 120 s. Data were analyzed with BiacoreTM Insight Software by curve fitting.

### Transfection of small interfering RNA

Predesigned small interfering RNAs (siRNAs) against PPARα and control scrambled siRNAs were synthesized by Hanbio Biotechnology Co. Ltd. The sequences of the PPARα siRNAs used were as follows: for PPARAα, the sequences of the sense and antisense siRNAs were 5′-GGGUUUAUAACUCGUGAAUTT-3′ and 5′-AUUCACGAGUUAUAAACCCTT-3′, respectively. For the negative control, the sense and antisense siRNAs used were 5′-UUCUCCGAACGUGUCACGUTT-3′ and 5′-ACGUGACACGUUCGGAGAATT-3′, respectively. The cells were then transfected with Opti-MEM and control or PPARα siRNA together with Lipo8000 transfection reagent for 24 h. After transfection, the culture medium was changed, and the cells were incubated with phytosphingosine for 48 h. The effectiveness of PPARα silencing was verified via western blotting.

### Statistical analysis

The statistical analyses were performed via IBM SPSS Statistics software (version 16.0) unless otherwise stated. The normal distribution assumption was tested with the Shapiro‒Wilk test and Q‒Q plots. The data from the human studies are presented as the means ± SDs. Comparisons of the basic characteristics were conducted with an independent Student’s *t*-test or one-way ANOVA with Tukey’s post hoc test for normally distributed data or the Wilcoxon rank-sum test for nonnormally distributed data. The categorical variables were analyzed with the χ^2^ test. For the animal, cell and germ culture studies, the sample sizes were estimated on the basis of previous studies, and no statistical test was used to predetermine the sample sizes. The animal data are presented as the means ± SEM, and all analyses were performed via GraphPad Prism 9.0.

## Supplementary information

Supplementary Information
