# Supplementary material for: A decrease in Flavonifractor plautii and its product, phytosphingosine, predisposes individuals with phlegm-dampness constitution to metabolic disorders
Source: Cell Discov. 2025 Mar 17;11:25. doi: 10.1038/s41421-025-00789-x (PMC11914097; doi:10.1038/s41421-025-00789-x)
Supplement: Supplementary file 1 — Supplementary Information [file 41421_2025_789_MOESM1_ESM.pdf]

Supplementary figures

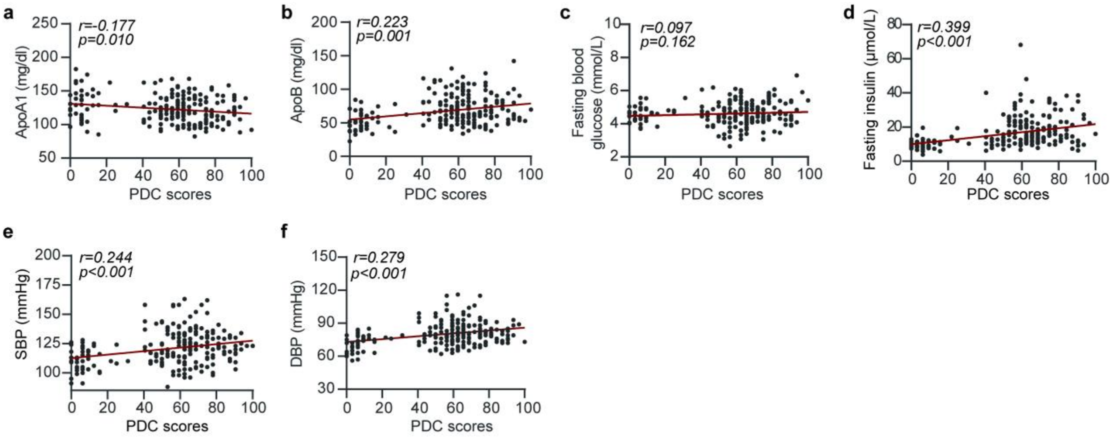

Fig. S1. Correlation analysis between PDC scores and partial metabolic indices in all subjects. a. Spearman correlations (two-tailed Spearman's rank test) between the PDC scores and the serum ApoA1 level. **b**. Spearman correlations (two-tailed Spearman's rank test) between the PDC scores and the serum ApoB level. **c**. Spearman correlations (two-tailed Spearman's rank test) between PDC scores and fasting blood glucose levels. **d**. Spearman correlations (two-tailed Spearman's rank test) between PDC scores and fasting insulin levels. **e**. Spearman correlations (two-tailed Spearman's rank test) between the PDC scores and SBP. **f**. Spearman correlations (two-tailed Spearman's rank test) between the PDC scores and DBP. ApoA1, serum apolipoprotein A1; ApoB, serum apolipoprotein B; SBP, systolic blood pressure; DBP, diastolic blood pressure.

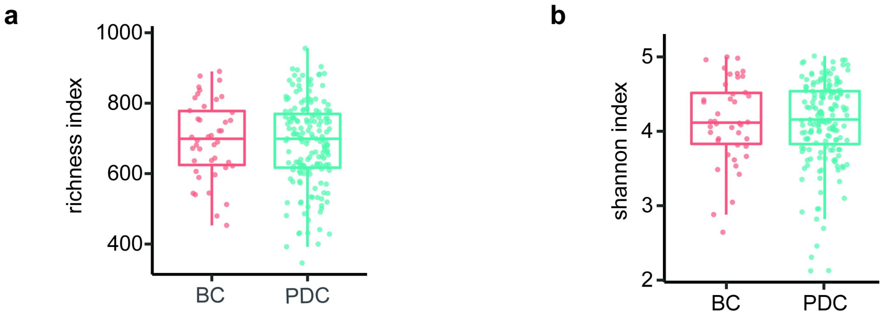

Fig. S2. Comparison of α diversity indices (richness index and Shannon index) between the BC group and PDC group. a. richness index; **b**. Shannon index. The differences were determined by the Wilcoxon rank-sum test. *, **, and *** indicate $p < 0.05$, $p < 0.01$, and $p < 0.001$, respectively.

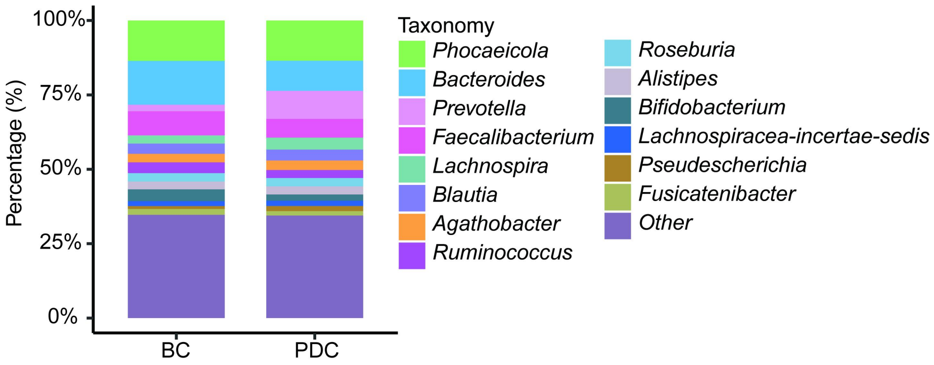

Fig. S3. Gut microbiota compositions in the BC group and PDC group at the genus level.

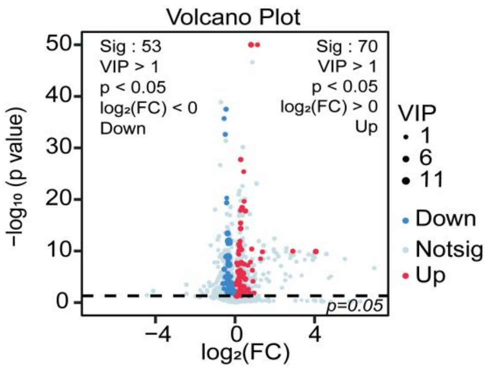

Fig. S4. Volcano plot of differentially abundant metabolites in the BC group and PDC group. P values adjusted by Benjamini–Hochberg < 0.1, absolute $\log_2$ fold change > 0.5, and variable importance for the projection (VIP) > 1 were considered differentially abundant metabolites. The x-axis represents the adjusted p value. The y-axis represents the $\log_2$ fold change.

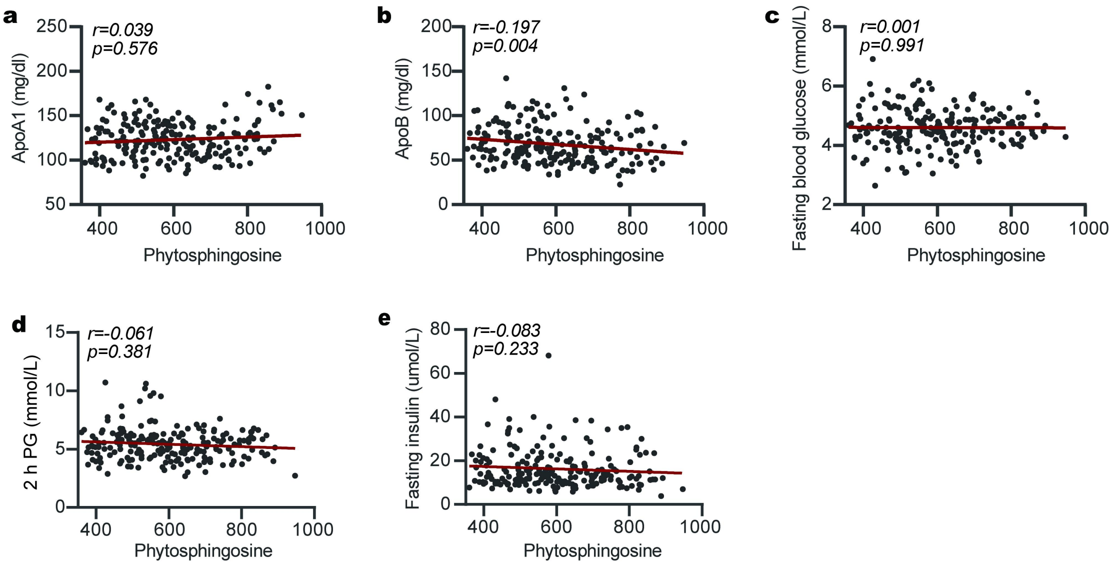

Fig. S5. Correlation analysis between phytosphingosine and metabolic indices. a. Spearman correlations (two-tailed Spearman's rank test) between serum phytosphingosine abundance and serum APOA1. **b**. Spearman correlations (two-tailed Spearman's rank test) between serum phytosphingosine abundance and serum APOB. **c**. Spearman correlations (two-tailed Spearman's rank test) between serum phytosphingosine abundance and fasting blood glucose. **d**. Spearman correlations (two-tailed Spearman's rank test) between serum phytosphingosine abundance and 2 h PG. **e**. Spearman correlations (two-tailed Spearman's rank test) between serum phytosphingosine abundance and fasting insulin. ApoA1, serum apolipoprotein A1; ApoB, serum apolipoprotein B; 2 h PG, 2 h postprandial blood glucose; SBP, systolic blood pressure; DBP, diastolic blood pressure.

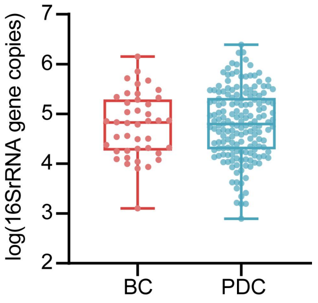

Fig. S6. Gene copies of 16S rRNA in the BC group and PDC group. The differences were determined using t tests. The data in the bar plot are presented as the means ± SDs. *, **, and *** indicate $p < 0.05$, $p < 0.01$, and $p < 0.001$, respectively.

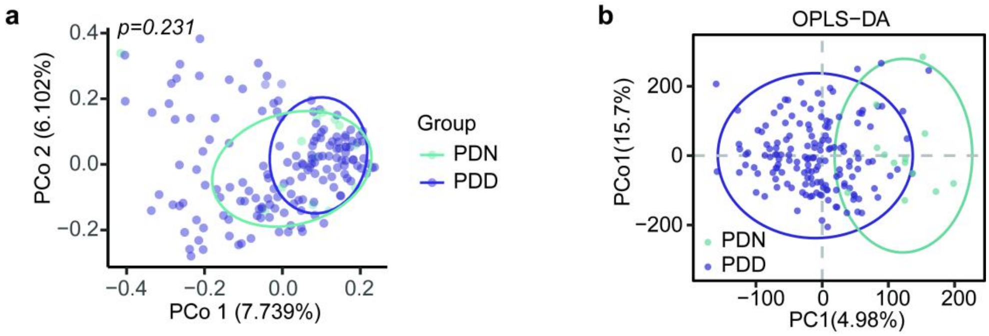

Fig. S7. PCoA plot of the gut microbiota and OPLS–DA plot of the serum metabolic profiles in the PDN group and PDD group. a. PCoA of the gut microbiota calculated from the Bray–Curtis distance in the PDN group and PDD group. p values were calculated with adonis via 6,000 permutations. **b**. OPLS–DA plot of the serum metabolites in the PDN group and PDD group. The p values of the model are calculated via 1,000 permutations.

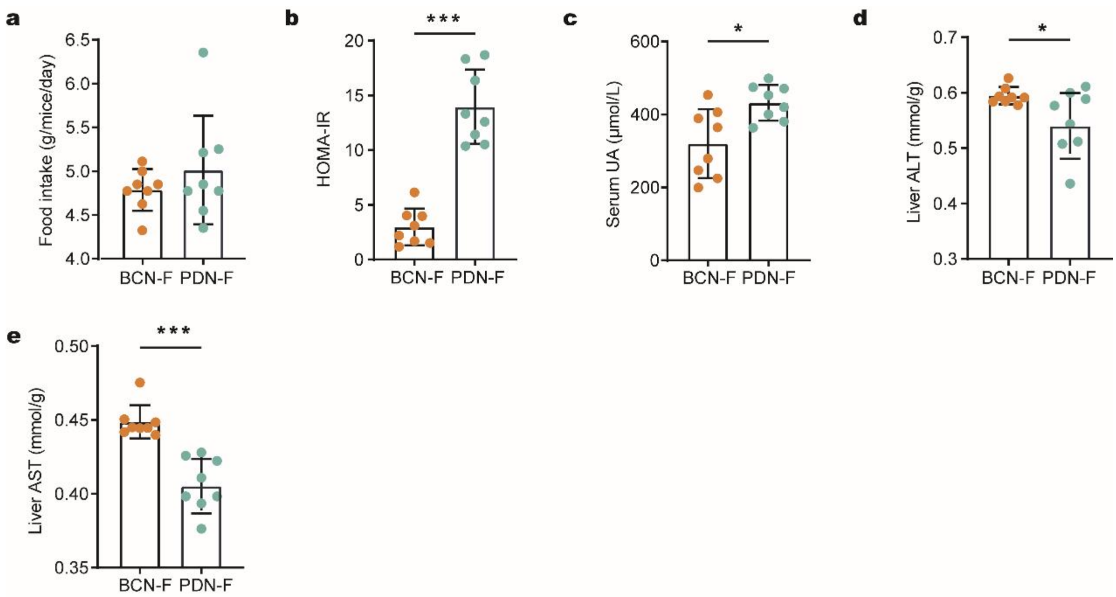

Fig. S8. Food intake (a), HOMA-IR (b), serum UA (c), liver ALT (d), liver AST (e) levels in the BCN-F group and PDN-F group. The differences were determined using t tests. The data in the bar plot are presented as the means ± SDs. *, **, and *** indicate $p < 0.05$, $p < 0.01$, and $p < 0.001$, respectively. HOMA-IR: Homeostasis Model Assessment-Insulin Resistance. UA, uric acid. In the BCN-F group, the antibiotic-treated mice received fecal slurry from the BCN group daily for 14

consecutive days and were fed a HFD for 5 weeks; in the PDN-F group, the antibiotic-treated mice received fecal slurry from the PDN group daily for 14 consecutive days and were fed a HFD for 5 weeks.

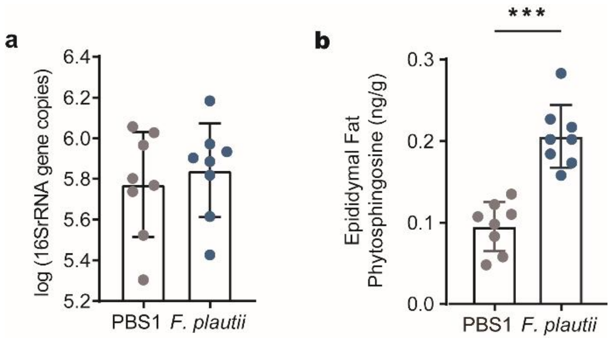

Fig. S9. Gene copies of 16S rRNA (a), and the levels of phytosphingosine in epididymal fat (b) in the PBS1 group and *F. plautii* group. The differences were determined using t tests. The data in the bar plot are presented as the means ± SDs. *, **, and *** indicate $p < 0.05$, $p < 0.01$, and $p < 0.001$, respectively. In the PBS1 group, the PDN-F mice were gavaged with PBS every day in animal experiment 2. In the *F. plautii* group, PDN-F mice were gavaged with 5×10^8 cfu *F. plautii* every day in animal experiment 2.

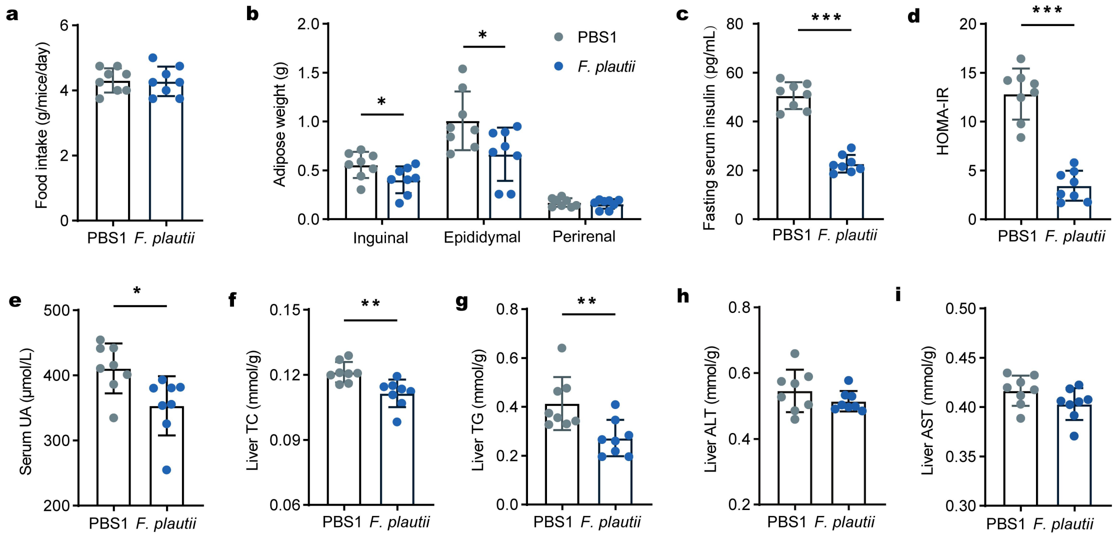

Fig. S10. Food intake (a), adipose weight (b), fasting serum insulin (c), HOMA-IR (d), serum uric acid (e), liver TC (f), liver TG (g), liver ALT (h), and liver AST (i) in the PBS1 group and *F. plautii* group. The differences were determined using t tests. The data in the bar plot are presented as the means ± SDs. *, **, and *** indicate $p < 0.05$, $p < 0.01$, and $p < 0.001$, respectively. In the PBS1

group, the PDN-F mice were gavaged with PBS every day in animal experiment 2. In the *F. plautii* group, PDN-F mice were gavaged with 5×10^8 cfu *F. plautii* every day in animal experiment 2.

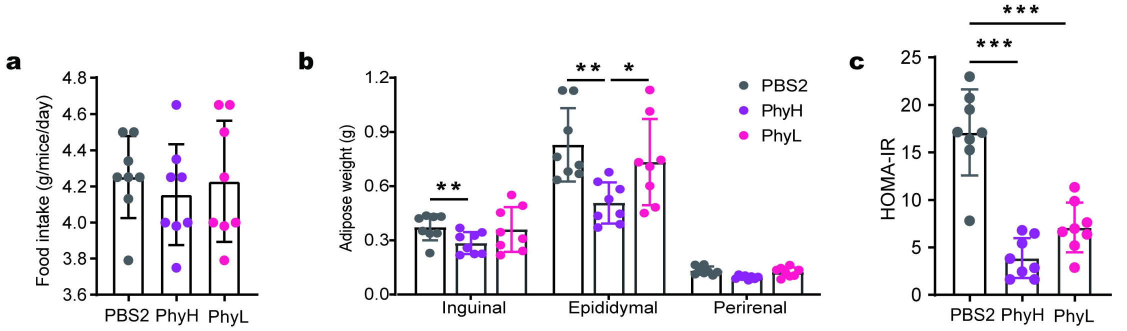

Fig. S11 Food intake (a), adipose weight (b), and HOMA-IR (c) in the PBS2, PhyH, and PhyL groups. Differences among groups were analyzed by one-way ANOVA with Tukey's post hoc test. The data in the bar plot are presented as the means ± SDs. *, **, and *** indicate $p < 0.05$, $p < 0.01$, and $p < 0.001$, respectively. HOMA-IR (Homeostasis Model Assessment of Insulin Resistance) = fasting serum glucose × fasting serum insulin / 22.5). In the PBS2 group, the PDN-F mice were gavaged with PBS every day in animal experiment 3. In the PhyH group, PDN-F mice were gavaged with 50 mg/kg phytosphingosine every day in animal experiment 3. In the PhyL group, PDN-F mice were gavaged with 25 mg/kg phytosphingosine every day in animal experiment 3.

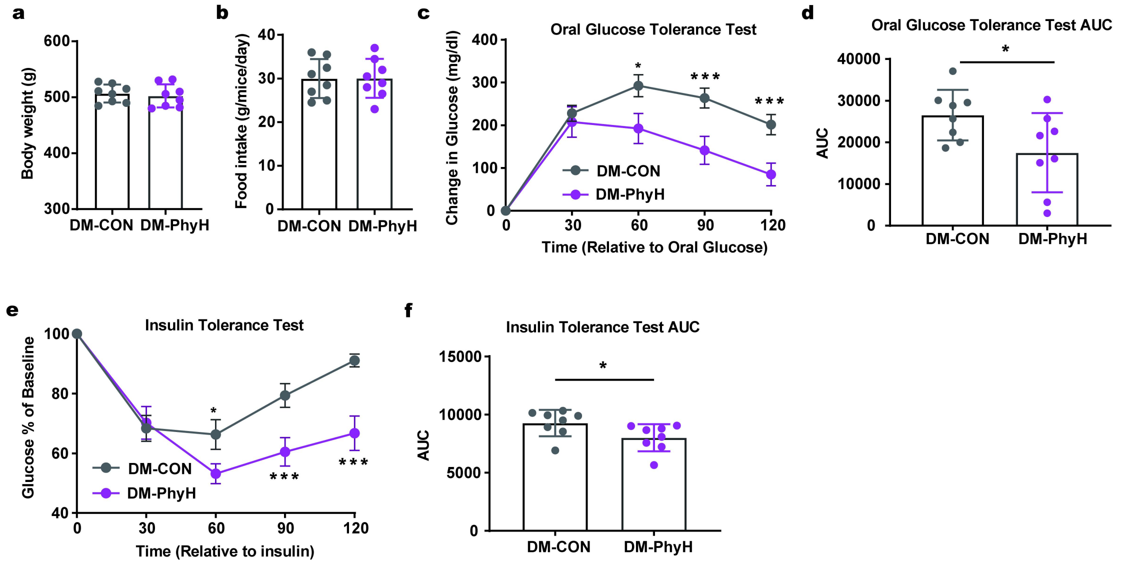

Fig. S12 Effects of phytosphingosine on insulin resistance in type 2 diabetic rats. **a**. Body weight. **b**. Food intake. **c**. GTT. **d.** GTT AUC. **e.** ITT. **f.** ITT AUC.

Differences were analyzed using t tests. The data in the bar plot are presented as the means ± SDs. *, **, and *** indicate $p < 0.05$, $p < 0.01$, and $p < 0.001$, respectively. GTT, glucose tolerance test; ITT, insulin tolerance test; AUC, Area Under Curve. In the DM-CON group, the type 2 diabetic rats were gavaged with PBS every day. In the DM-PhyH group, the type 2 diabetic rats were gavaged with 50 mg/kg phytosphingosine every day.

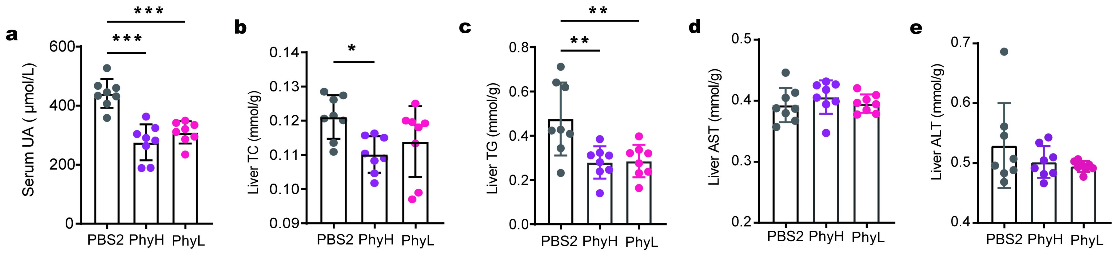

Fig. S13 Serum UA (a), liver TC (b), liver TG (c), liver AST (d), and liver ALT (e) in the PBS2, PhyH, and PhyL groups. Differences among groups were analyzed by one-way ANOVA with Tukey's post hoc test. The data in the bar plot are presented as the means ± SDs. *, **, and *** indicate $p < 0.05$, $p < 0.01$, and $p < 0.001$, respectively. In the PBS2 group, the PDN-F mice were gavaged with PBS every day in animal experiment 3. In the PhyH group, PDN-F mice were gavaged with 50 mg/kg phytosphingosine every day in animal experiment 3. In the PhyL group, PDN-F mice were gavaged with 25 mg/kg phytosphingosine every day in animal experiment 3.

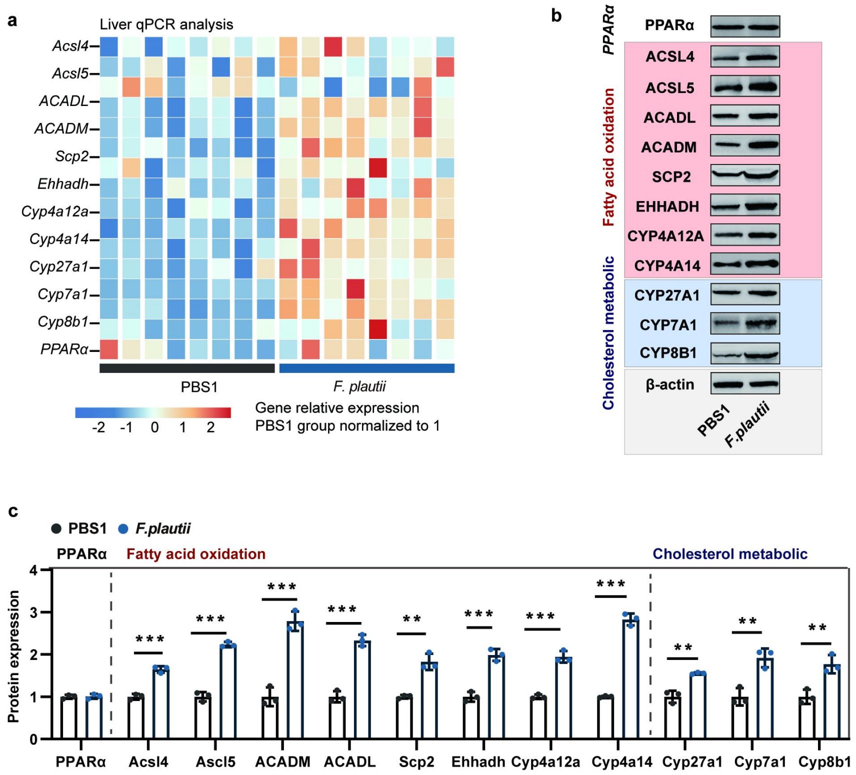

Fig. S14 Expression and immunoblot analysis of hepatic PPARα and downstream genes in the PBS1 group and *F. plautii* group. a. Heatmap of hepatic PPARα and downstream gene relative mRNA expression in the PBS1 group and *F. plautii* group. The relative gene expression in the PBS group was normalized to 1. Five biological replicates were performed for each group. **b, c.** Immunoblot densitometry analysis of hepatic PPARα and downstream genes in the PBS1 group and *F. plautii* group. Differences were analyzed using t tests. The data in the bar plot are presented as the means ± SDs. *, **, and *** indicate $p < 0.05$, $p < 0.01$, and $p < 0.001$, respectively. In the PBS1 group, the PDN-F mice were gavaged with PBS every day in animal experiment 2. In the *F. plautii* group, PDN-F mice were gavaged with 5×10^8 cfu *F. plautii* every day in animal experiment 2.

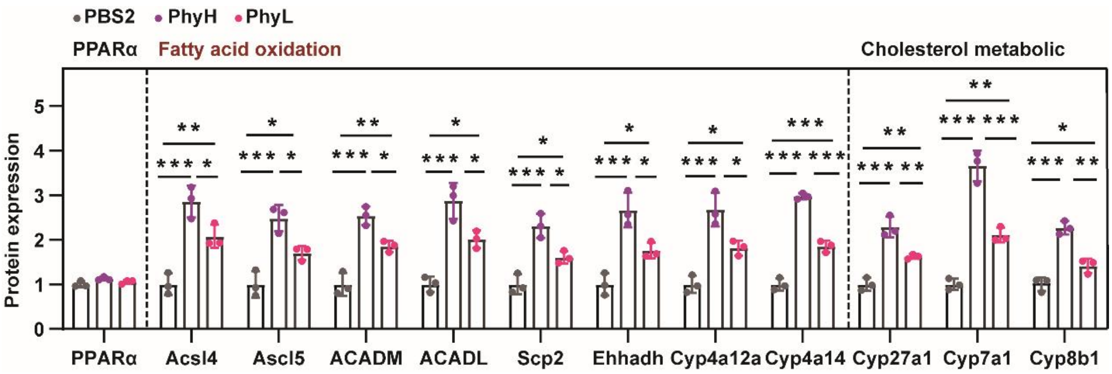

Fig. S15. Immunoblot densitometry analysis of hepatic PPARα and downstream genes in the PBS2, PhyH, and PhyL groups. Differences among groups were analyzed by one-way ANOVA with Tukey's post hoc test. The data in the bar plot are presented as the means ± SDs. *, **, and *** indicate $p < 0.05$, $p < 0.01$, and $p < 0.001$, respectively. In the PBS2 group, the PDN-F mice were gavaged with PBS every day in animal experiment 3. In the PhyH group, PDN-F mice were gavaged with 50 mg/kg phytosphingosine every day in animal experiment 3. In the PhyL group, PDN-F mice were gavaged with 25 mg/kg phytosphingosine every day in animal experiment 3.

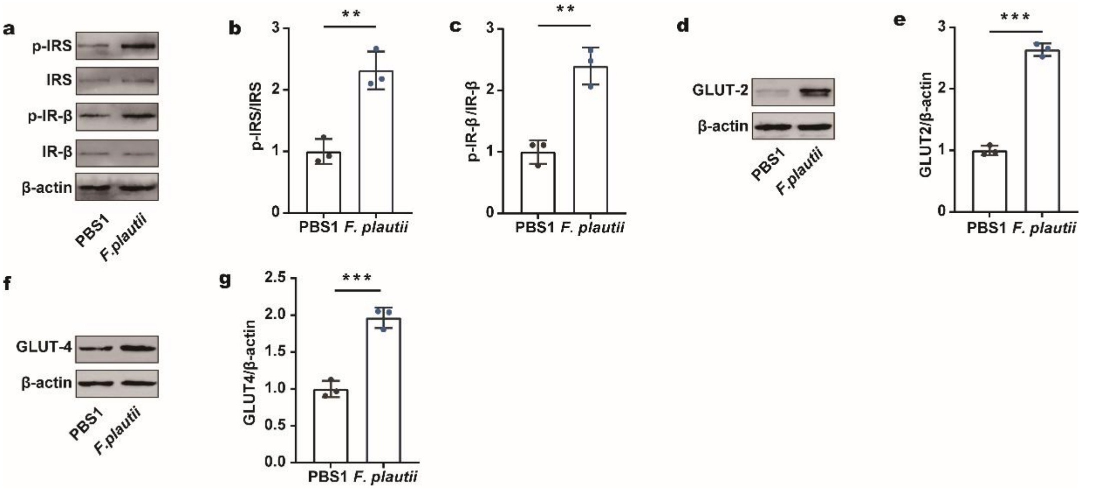

Fig. S16. Expression and immunoblot analysis of glucose metabolism related proteins in the PBS1 group and *F. plautii* group. a, b, c. Immunoblot densitometry analysis of hepatic p-IRS/IRS, and p-IRS/IRS in the PBS1 group and *F. plautii* group. **d**, **e**. Immunoblot densitometry analysis of hepatic GLUT-2/β-actin in the PBS1 group and *F. plautii* group. **f**, **g**. Immunoblot densitometry analysis of epididymal fat GLUT-4/β-actin in the PBS1 group and *F. plautii* group. Differences were analyzed

using t tests. The data in the bar plot are presented as the means ± SDs. *, **, and *** indicate $p < 0.05$, $p < 0.01$, and $p < 0.001$, respectively. In the PBS1 group, the PDN-F mice were gavaged with PBS every day in animal experiment 2. In the *F. plautii* group, PDN-F mice were gavaged with 5×10^8 cfu *F. plautii* every day in animal experiment 2.

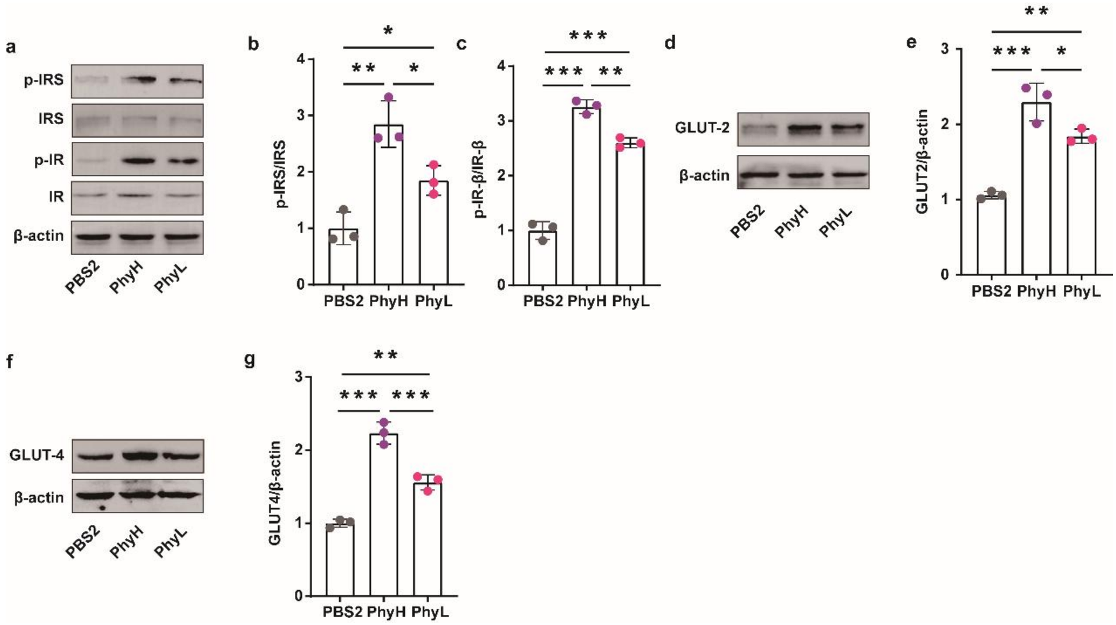

Fig. S17. Expression and immunoblot analysis of glucose metabolism related proteins in the PBS2, PhyH, and PhyL groups. a, b, c. Immunoblot densitometry analysis of hepatic p-IRS/IRS, and p-IRS/IRS in the PBS2, PhyH, and PhyL groups. **d, e.** Immunoblot densitometry analysis of hepatic GLUT-2/β-actin in the PBS2, PhyH, and PhyL groups. **f, g.** Immunoblot densitometry analysis of epididymal fat GLUT-4/β-actin in the PBS2, PhyH, and PhyL groups. Differences among groups were analyzed by one-way ANOVA with Tukey's post hoc test. The data in the bar plot are presented as the means ± SDs. *, **, and *** indicate $p < 0.05$, $p < 0.01$, and $p < 0.001$, respectively. In the PBS2 group, the PDN-F mice were gavaged with PBS every day in animal experiment 3. In the PhyH group, PDN-F mice were gavaged with 50 mg/kg phytosphingosine every day in animal experiment 3. In the PhyL group, PDN-F mice were gavaged with 25 mg/kg phytosphingosine every day in animal experiment 3.

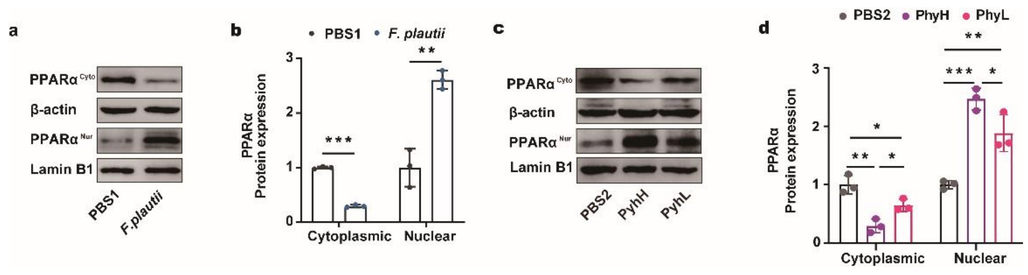

Fig. S18. **Expression and immunoblot analysis of PPARα in epididymal fat. a, b.** Immunoblot densitometry analysis of epididymal fat PPARα in the PBS1 group and *F. plautii* group. Differences (**b**) were analyzed using t tests. **c, d.** Immunoblot densitometry analysis of epididymal fat PPARα in the PBS2, PhyH, and PhyL groups. Differences (**d**) were analyzed by one-way ANOVA with Tukey's post hoc test. The data in the bar plot are presented as the means ± SDs. *, **, and *** indicate $p < 0.05$, $p < 0.01$, and $p < 0.001$, respectively. In the PBS1 group, the PDN-F mice were gavaged with PBS every day in animal experiment 2. In the *F. plautii* group, PDN-F mice were gavaged with 5×10^8 cfu *F. plautii* every day in animal experiment 2. In the PBS2 group, the PDN-F mice were gavaged with PBS every day in animal experiment 3. In the PhyH group, PDN-F mice were gavaged with 50 mg/kg phytosphingosine every day in animal experiment 3. In the PhyL group, PDN-F mice were gavaged with 25 mg/kg phytosphingosine every day in animal experiment 3.

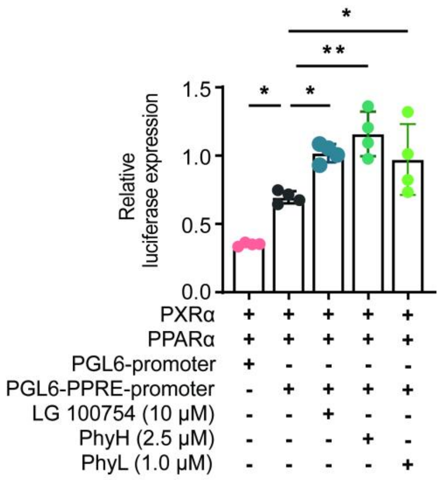

Fig. S19. PPARα transcriptional activation of phytosphingosine. Differences among groups were analyzed by one-way ANOVA with Tukey's post hoc test. The data in the bar plot are presented as the means ± SDs. *, **, and *** indicate p <

0.05, p < 0.01, and p < 0.001, respectively.

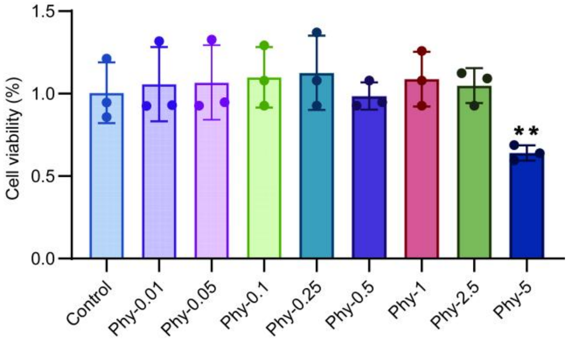

Fig. S20. Effects of phytosphingosine on HepG2 cell viability. Differences were analyzed using one-way ANOVA with Tukey's post hoc test. The data in the bar plot are presented as the means ± SDs. * $p < 0.05$, ** $p < 0.01$, and *** $p < 0.001$ represent significant differences compared with the control group.

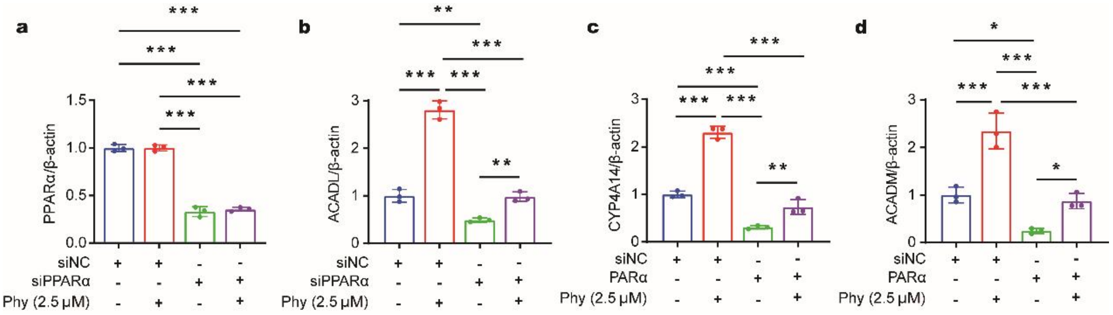

Fig. S21. Densitometric analysis of PPARα (a), ACADL (b), CYP4A14 (c), and ACADM (d) after PPARα was knocked down in insulin-induced HepG2 cells treated with phytosphingosine. n=3. Differences among groups were analyzed by one-way ANOVA with Tukey's post hoc test. The data in the bar plot are presented as the means ± SDs. *, **, and *** indicate $p < 0.05$, $p < 0.01$, and $p < 0.001$, respectively.

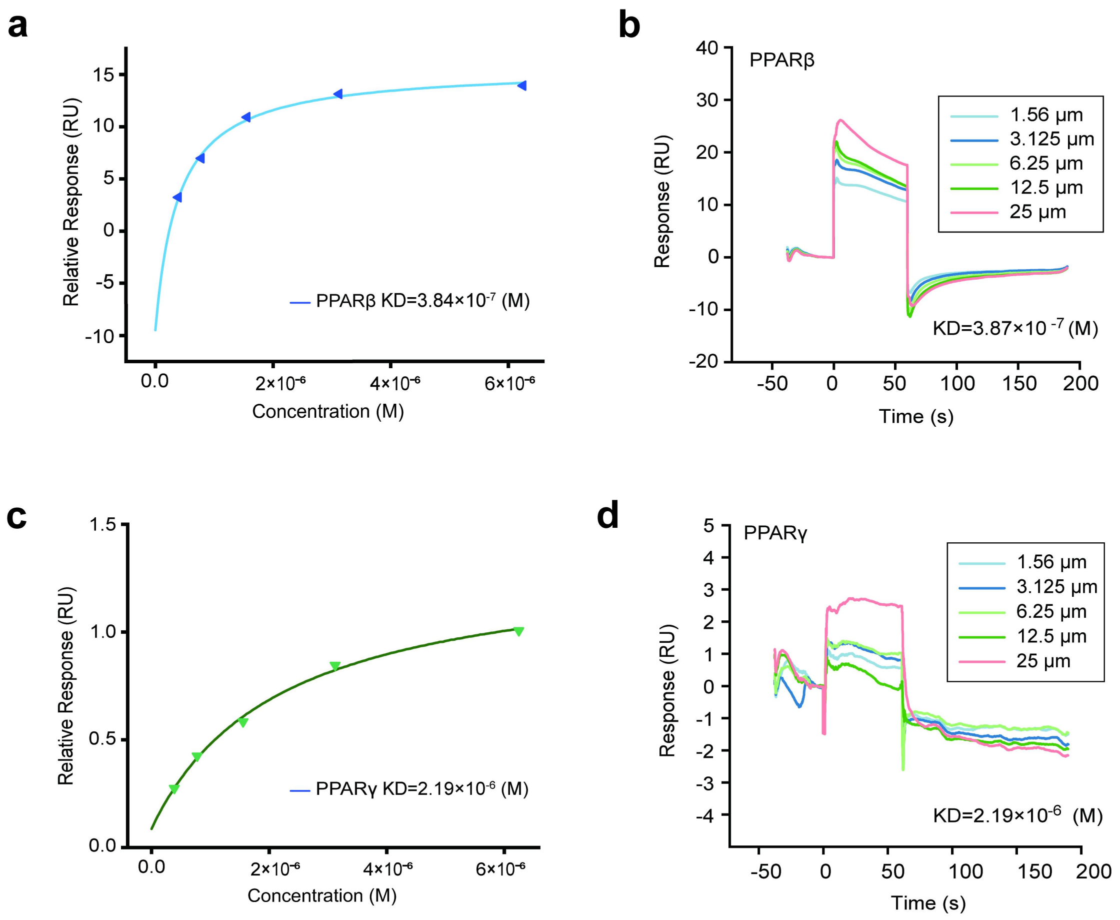

Fig. S22. SPR binding analysis of phytosphingosine to PPARβ (**a, b**) or PPARγ (**c, d**).

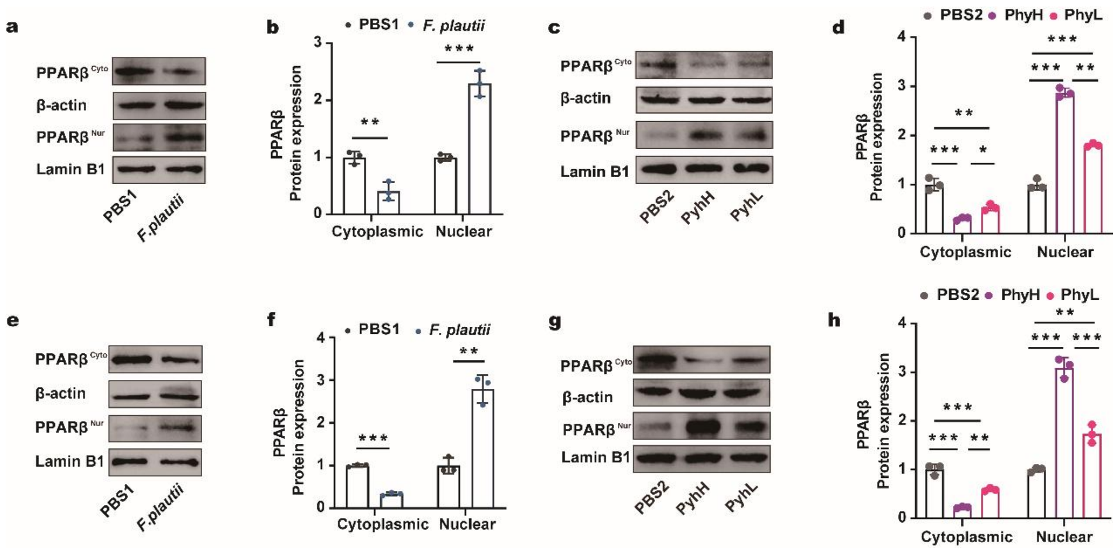

Fig. S23. Expression and immunoblot analysis of hepatic and epididymal fat PPARβ. a, b. Immunoblot and densitometry analysis of hepatic PPARβ in the PBS1 group and *F. plautii* group. **c, d**. Immunoblot and densitometry analysis of hepatic PPARβ in the PBS2, PhyH, and PhyL groups. **e, f**. Immunoblot and densitometry

analysis of epididymal fat PPARβ in the PBS1 group and *F. plautii* group. **g, h**. Immunoblot and densitometry analysis of epididymal fat PPARβ in the PBS2, PhyH, and PhyL groups. Differences (**b, f**) were analyzed using t tests. Differences (**d, h**) were analyzed by one-way ANOVA with Tukey's post hoc test. The data in the bar plot are presented as the means ± SDs. *, **, and *** indicate $p < 0.05$, $p < 0.01$, and $p < 0.001$, respectively. In the PBS1 group, the PDN-F mice were gavaged with PBS every day in animal experiment 2. In the *F. plautii* group, PDN-F mice were gavaged with 5×10^8 cfu *F. plautii* every day in animal experiment 2. In the PBS2 group, the PDN-F mice were gavaged with PBS every day in animal experiment 3. In the PhyH group, PDN-F mice were gavaged with 50 mg/kg phytosphingosine every day in animal experiment 3. In the PhyL group, PDN-F mice were gavaged with 25 mg/kg phytosphingosine every day in animal experiment 3.

Supplementary tables

Table S1 Clinical characteristics of subjects in this study

**Characteristics**	**BC (n=42)**	**PDC (n=167)**	***p*-value**
Age (years)	27.5±6.26	34.75±8.61	< 0.001
Sex (Male/Female)	15/27	87/80	0.166
PDC score	7.29±6.46	65.77±13.7	< 0.001
BMI (kg/m^2)	21.43±1.68	28.48±4.2	< 0.001
Weight (kg)	59.84±7.64	80.86±15.87	< 0.001
Waist circumference (cm)	75.91±6.02	96.88±10.85	< 0.001
Hip circumference (cm)	93.05±3.96	105.55±8.12	< 0.001
Waist-hip ratio	0.77±0.06	0.90±0.08	0.008
TC (mmol/L)	4.2±0.58	4.83±0.89	< 0.001
TG (mmol/L)	0.69±0.25	1.66±1.23	< 0.001
HDLC (mmol/L)	1.39±0.22	1.14±0.24	< 0.001
LDLC (mmol/L)	2.13±0.51	2.82±0.72	< 0.001
ApoA1 (mg/dl)	130.09±23	121.25±19.03	0.011

ApoB (mg/dl)	52.61±14.3	71.66±21.2	< 0.001
Lpa (mg/dl)	11.56±12.81	9.62±10.58	0.310
Fast blood glucose (mM)	4.53±0.44	4.62±0.71	0.440
2 h PG (mM)	4.79±1.02	5.58±1.41	0.001
Insulin (IU/mL)	10.32±3.2	17.8±9.11	< 0.001
HBA1C%	5.17±0.31	5.38±0.42	0.003
Uric acid (μmol/L)	286.79±65.34	366.06±88.27	< 0.001
SBP (mmHg)	111.67±9.89	122.84±14.65	< 0.001
DBP (mmHg)	72.1±7.44	81.89±10.3	< 0.001
ALT (U/L)	15.6±7.57	26.74±16.66	< 0.001
AST (U/L)	18.07±4.53	21.1±9.34	0.043
Cr (μmol/L)	62.33±13.74	67.28±16.88	0.080

Continuous variables were presented as the means ± standard deviations. Categorical variables were presented as ratios. Differences in clinical characteristics between BC subjects and PDC subjects were compared by using Student's *t*-test for normal continuous variables, the Wilcoxon rank-sum test for nonnormal continuous variables, and the chi-square test or Fisher's exact test for categorical variables. Statistical significance was defined by $P < 0.05$ (two-tailed), BC, Balanced constitution; PDC, Phlegm dampness constitution.

Table S2 Characterization of samples for metagenomic sequencing

Characteristics	BC	PDC	p-value

N	10	40	
Sex (Female/Male)	5/5	18/22	0.106
Age (years)	29.3±6.4	33.83±9.02	0.143
PDC score	3.44±2.74	68.36±12.32	<0.001
BMI (kg/m^2)	20.84±1.48	28.63±4.17	<0.001
Weight (kg)	59.54±7.5	80.81±16.71	<0.001
Waist circumference(cm)	74.4±5.36	96.8±11.59	<0.001
Hip circumference(cm)	91.7±2.54	105.94±9.34	<0.001
TC (mmol/L)	3.79±0.54	5.08±1.03	<0.001
TG (mmol/L)	0.66±0.28	1.74±1.29	0.011
HDL (mmol/L)	1.33±0.22	1.12±0.22	0.012
LDL (mmol/L)	1.92±0.48	3.03±0.82	<0.001
ApoA1 (mg/dl)	132.37±25.74	120.86±19.21	0.120
ApoB (mg/dl)	48.81±15.52	76.55±21.78	<0.001
Lpa (mg/dl)	11.03±11.49	10.73±10.89	0.938
Fast blood glucose (mM)	4.58±0.51	4.86±0.68	0.229

2 h PG (mM)	4.96±0.82	6±1.72	0.069
INS (IU/mL)	11.03±2.33	19.67±11.24	0.020
HBA1C%	5.24±0.33	5.51±0.57	0.164
UA (µmol/L)	296.6±84.31	354.35±91.94	0.066
SBP (mmHg)	109.3±10.4	122.85±13.93	0.006
DBP (mmHg)	67±7.59	81.58±8.84	<0.001
ALT (U/L)	20.7±10.96	26.78±16.02	0.264
AST (U/L)	22.4±5.1	20.5±8.31	0.494
Cr (µmol/L)	63.6±12.48	62.45±19.27	0.859

Continuous variables were presented as the means ± standard deviations. Categorical variables were presented as ratios. Differences in clinical characteristics between BC and PDC groups were compared by using Student's t-test for normal continuous variables, the Wilcoxon rank-sum test for nonnormal continuous variables, and the chi-square test for categorical variables. Statistical significance was defined by $p < 0.05$ (two-tailed), BC, Balanced constitution; PDC. Phlegm-dampness constitution

Table S3. Characteristics of the donors for fecal transplantation

**Characteristics**	**BC subjects with normal metabolic indices**	**PDC subjects with normal metabolic indices**

	A006	A010	A019	A033	A043	A082	A099	A136	A156	A170
**sex(Male/Female)**	**Female**	**Female**	**Female**	**Male**	**Male**	**Male**	**Female**	**Male**	**Male**	**Female**
Age (years)	27	23	25	32	27	36	23	48	33	43
PDC score	3.125	3.13	3.13	6.25	15.63	78.13	75.00	59.38	68.75	40.63
BMI (kg/m2)	18.87970615	19.27	20.30	21.38	22.34	25.55	23.49	23.93	26.21	23.62
Weight (kg)	51.4	51.5	59	64	70	80.5	62.4	73.3	78	62
Waist circumference (cm)	70	71	72	72	88	93	88	91	96	84
Hip circumference (cm)	88	89	94	91	97	107	99	101	110	97
TC (mmol/L)	3.03	4.2	3.61	2.91	3.63	3.63	4.54	4.15	3.67	4.39
TG (mmol/L)	0.46	0.63	0.48	0.84	0.86	0.53	0.69	0.55	1.06	0.75
HDLC (mmol/L)	1.52	1.62	1.49	1.03	1.02	1.22	1.82	1.71	1.21	1.37
LDLC (mmol/L)	1.14	1.87	1.54	1.5	2.13	1.88	2.55	1.71	1.74	2.28
ApoA1 (mg/dl)	146.4	161.7	116.4	107.3	85.1	114.8	140.4	155.3	100	126.6
ApoB (mg/dl)	32.7	39.3	37.5	38.7	51	38.1	44.2	57.7	61.1	46.3
Lpa (mg/dl)	2.1	0.8	2.5	13	7.5	10.3	1.6	11.4	6.6	27.1

Fasting blood glucose (mM)	5.05	4.28	4.68	4.96	3.82	5.72	4.4	5.63	4.19	4.76
2 h PG (mM)	5.91	4.47	3.04	5.41	3.71	4.31	4.87	5.52	5.77	7.45
Insulin (IU/mL)	11.175	11.479	8.651	8.04	10.021	11.717	9.752	10.731	17.643	6.2
HBA1C%	5.8	5.1	4.6	5.3	4.9	5.1	5.1	5.5	4.9	6
UA (μmol/L)	246	192	149	294	275	246	265	351	274	217
SBP (mmHg)	99	104	105	105	116	100	113	139	109	119
DBP (mmHg)	65	72	62	57	75	70	78	88	80	73
ALT (U/L)	14	15	10	16	21	14	9	19	8	12
AST (U/L)	20	21	12	22	17	16	14	17	15	18
Cr (μmol/L)	51	55	56	86	84	84	50	67	72	50

NOTE: Abbreviations: BC, Balanced constitution; PDC, Phlegm dampness constitution.

Table S4 Primer sequences for PCR

Name	Sequence (5'-3')	Types	PCR Products (bp)
16S	TCCTACGGGAGGCAGCAGT	Forward Primer	450

	GGACTACCAGGGTATCTAATCCTGTT	Reverse Primer	
F. plautii	TTGTCGGGGACGAAACAAAT	Forward Primer	204
	CTCCAGCACTCAAGAACTAC	Reverse Primer	
Scp2	TGCCAGGAGATGCTATGAGAAGTC	FORWARD	107
	CCAGTGCTTCGTAAGTGATGAGTTC	REVERSE	
Gapdh	GCAAATTCAACGGCACAGTCAAG	FORWARD	80
	TCGCTCCTGGAAGATGGTGATG	REVERSE	
Ehhadh	AGTCGTTGTTGGTAACTGCTATGG	FORWARD	105
	ACATCCTCTGGCTTACTACCTTCC	REVERSE	
Cyp27a1	CATCTTCTCTACCACCTTGCCTTG	FORWARD	139
	AGACTGAGTTCTGGAACATGATTGC	REVERSE	
Cyp8b1	GACAGCCTATCCTTGGTGATGC	FORWARD	141
	GTCCTTGGTGTAGCCGAATAAGC	REVERSE	
Cyp7a1	AGGTCTCTGAACTGATCCGTCTAC	FORWARD	80
	CGTCTTAGCCTTCTCCATGTCATC	REVERSE	
Cyp4a32	CTCTAAGCCTGACCCGATTTGC	FORWARD	127
	GCCTTGAGTAGCCATTTCCTGTG	REVERSE	

Cyp4a31	CTCTAAGCCTGACCCGATTTGC	FORWARD	127
	GCCTTGAGTAGCCATTTCCTGTG	REVERSE	
Cyp4a12b	GTGTCCTCTAATGGCTGCTTGG	FORWARD	101
	TCCTCATCCTGAAGTTGACTCCTC	REVERSE	
Cyp4a12a	CAGCACCGACGAATGTTGACC	FORWARD	93
	ATCCAGCATTACACGAACAGAGTC	REVERSE	
Cy4a10	CTCTAAGCCCAACCCGATTTGC	FORWARD	126
	CCTTGAGTAGCCATTGCCTGTG	REVERSE	
Ascl5	TTGAGAGGCTTGTCCAGGGTATC	FORWARD	136
	TTAAGGAGTCGAGGCACTGTAGG	REVERSE	
Acsl4	GCCGCCATCAGCTACATCAAG	FORWARD	129
	CTAGCTACGCTCCTCAGACTCG	REVERSE	
Acaa1b	GTTTCACGGCAGAAGCAGGATG	FORWARD	97
	CAGGCACAATCTCAGCATGGAAG	REVERSE	
PPARα	CGATGCTGTCCTCCTTGATGAAC	FORWARD	105
	TGTCACAGAACGGCTTCCTCAG	REVERSE	
ACADM	AATTACCGAAGAGTTGGCGTATGG	FORWARD	145

	CATCATTGGCTGCTCCGTCATC	REVERSE	
ACADL	CTTCAGCCTCCACTCAGATATTGTC	FORWARD	101
	TACACTTGCCCGCCGTCATC	REVERSE	

Supplementary Methods

Section 1： Classification and Determination of Constitution in TCM (Version: ZYYXH/T157-2009)

• TCM Constitution Scale

This questionnaire aims to survey your constitution and to subsequently provide a reference for future health management and clinical diagnosis. Please read every question carefully and choose the most suitable response based on your actual situation or experience in the past year. If you are unsure of the answer to a specific question, choose the answer that is most similar to your actual situation. Make sure that you answer all the questions based on your situation in the past year, (excluding the effect of drugs) and give only one answer to each question.

Explain:【No】Never happened in the past year.【Slightly】Occasionally happened in the past year.【Sometimes】Sometimes it happened, but no regular pattern. 【Often】 It happened most of the time in the past year. 【All the time】 It happened all the time in the past year.

**Experience/condition in the past year**	**No**	**Slightly**	**Sometimes**	**Often**	**All the time**
(1) Were you energetic?	1	2	3	4	5
(2) Did you get tired easily?	1	2	3	4	5
(3) Did you experience shortness of breath?	1	2	3	4	5
(4) Did you get palpitations?	1	2	3	4	5
(5) Did you get dizzy easily or become dizzy when standing up?	1	2	3	4	5
(6) Did you prefer quietness and not like to talk?	1	2	3	4	5
(7) Was your voice weak when talking?	1	2	3	4	5
(8) Did you feel in low spirits and depressed?	1	2	3	4	5
(9) Did you easily feel anxious and	1	2	3	4	5

worried?					
(10) Did you feel overly sensitive, vulnerable or emotionally upset?	1	2	3	4	5
(11) Were you easily scared or frightened?	1	2	3	4	5
(12) Did you experience distention in the underarm or breast?	1	2	3	4	5
(13) Did you feel chest or abdominal stuffiness?	1	2	3	4	5
(14) Did you sigh without reason?	1	2	3	4	5
(15) Did your body feel heavy or lethargic?	1	2	3	4	5
(16) Did the palms of your hands or soles of your feet feel hot?	1	2	3	4	5
(17) Did your hands or feet feel cold or clammy?	1	2	3	4	5
(18) Did you feel cold easily in your abdomen, back, lower back or knees?	1	2	3	4	5
(19) Were you sensitive to cold and tended to wear more clothes than others?	1	2	3	4	5
(20) Did your body and face feel hot?	1	2	3	4	5
(21) Did you feel more vulnerable to the cold than others (winter coldness, air conditioners, fans, etc.)?	1	2	3	4	5
(22) Did you catch colds more easily than others?	1	2	3	4	5
(23) Did you sneeze even when you did not have a cold?	1	2	3	4	5
(24) Did you have a runny or stuffy nose	1	2	3	4	5

even when you did not have a cold?					
(25) Did you cough due to seasonal changes, temperature changes, or unpleasant odors?	1	2	3	4	5
(26) Did you sweat easily when your physical activity increased slightly?	1	2	3	4	5
(27) Did you forget things easily?	1	2	3	4	5
(28) Did you have an excessively oily forehead and/or T-zone?	1	2	3	4	5
(29) Were your lips redder than in the past?	1	2	3	4	5
(30) Did you have allergies? (e.g. medicine, food, odors, pollen, pet dander, or during seasonal or weather change etc.) Experience/condition in the past year No Slightly Sometimes Often All the time	1	2	3	4	5
(31) Did you get hives/urticaria easily?	1	2	3	4	5
(32) Did your skin have purpura (purple spots, ecchymosis) due to allergies?	1	2	3	4	5
(33) Did black or purple bruises appear on your skin for no reason?	1	2	3	4	5
(34) Did your skin turn red and show traces when you scratched it?	1	2	3	4	5
(35) Did your skin or lips feel dry?	1	2	3	4	5
(36) Did you have visible capillary (thread) veins on your cheeks?	1	2	3	4	5
(37) Did you feel	1	2	3	4	5

pain somewhere in your body?					
(38) Did you experience hot flashes?	1	2	3	4	5
(39) Did your nose or your face feel greasy, oily, or shiny?	1	2	3	4	5
(40) Did you have a dark face or get brown spots easily?	1	2	3	4	5
(41) Did you get acne or sores easily?	1	2	3	4	5
(42) Did you have upper eyelid swelling?	1	2	3	4	5
(43) Did you get dark circles under the eyes easily?	1	2	3	4	5
(44) Did your eyes feel dry and you used eye drops?	1	2	3	4	5
(45) Were your lips darker, more blue or purple than usual?	1	2	3	4	5
(46) Did you often feel parched and need to drink water?	1	2	3	4	5
(47) Did your throat feel strange (i.e., as if something was stuck or there was a lump in your throat)?	1	2	3	4	5
(48) Did you have a bitter or strange taste in your mouth?	1	2	3	4	5
(49) Did your mouth feel sticky?	1	2	3	4	5
(50) Was your abdomen flabby?	1	2	3	4	5
(51) Did you have an abundance of phlegm, especially in your throat?	1	2	3	4	5
(52) Did you feel uncomfortable when you drank or ate something cold, or did you avoid to drinking or eating cold items?	1	2	3	4	5
(53) Could you	1	2	3	4	5

adapt yourself to external natural or social environment changes?					
(54) Did you easily experience insomnia?	1	2	3	4	5
(55) Did you easily contract diarrhea when you were exposed to cold or ate (or drank) something cold?	1	2	3	4	5
(56) Did you pass sticky stools and/or feel that your bowel movement was incomplete?	1	2	3	4	5
(57) Did you get constipated easily or have dry stools?	1	2	3	4	5
(58) Did your tongue have a thick coating?	1	2	3	4	5
(59) Did your urethral canal feel hot when you urinated, or did your urine have a dark color?	1	2	3	4	5
(60) Was your vaginal discharge yellowish (only for female interviewees)?	1	2	3	4	5
(60) Was your scrotum always wet (only for male interviewees)?	1	2	3	4	5

• **Number of items corresponding to constitutional types**

**Constitutional type**	**Number of items**
balanced constitution	1, 2*, 7*, 8*, 21*, 27*, 53, 54*
qi-deficiency constitution	2, 3, 4, 5, 6, 7, 22, 26
yang-deficiency constitution	17, 18, 19, 21, 22, 52, 55
yin-deficiency constitution	16, 20, 29, 35, 38, 44, 46, 57
phlegm-dampness constitution	13, 15, 28, 42, 49, 50, 51, 58
dampness-heat constitution	5, 39, 41, 48, 56, 60 (for male), 60(for femal)
blood stasis constitution	27, 33, 36, 37, 40, 43, 45
qi stagnation constitution	8, 9, 10, 11, 12, 14, 47

inherited special constitution	23, 24, 25, 30, 31, 32, 34

• Determination method of constitution type

Answer all the questions in the TCM Constitution Scale (see below). Each item is graded on a scale of 1-5. Calculate the original score and transformation score, and judge the physique type according to the standard.

Original score = the scores of each item are added up (The marked * will be scored reversely when judging the balanced constitution).

Conversion score = [(original score-number of items) / (number of items ×4)] ×100.

• Criteria for determination

The balanced constitution is normal constitution, and the other eight constitutions are pathological constitution. See the table below for the criteria.

Table of criteria for judging balanced constitution and pathological constitutions

**Constitutional type**	**Requisite**	**Judgement result**
**Balanced constitution**	The conversion score ≥60 points	Yes
	The transformation score of other 8 pathological constitutions is less than 30.	
	The conversion score ≥60 points	Inclined to yes
	The transformation score of other 8 pathological constitutions is less than 40.	
	Those who do not meet the above conditions	No
**Pathological constitution**	The conversion score ≥40 points	Yes
	The conversion score is 30 ~ 39	Inclined to yes
	The Conversion score < 30 points	No

Section 2：Definitions of Metabolic disorders

Metabolic disorders represent a group of disorders with the clustering of various inter-related pathological conditions combining obesity, dyslipidemia, and diabetes. In our study, we assessed the participants for obesity, dyslipidemia, hyperuricemia, prediabetes, and hypertension. Obesity: body mass index (BMI) ⩾ 28 kg/m^2; Overweight: BMI ⩾ 24 kg/m^2; Dyslipidemia: at least one of fasting total cholesterol (TC) ⩾ 5.2 mmol/L, triglycerides (TG) ⩾ 1.7 mmol/L, Low-density lipoprotein cholesterol (LDL-C) ⩾ 3.4 mmol/L, high-density lipoprotein cholesterol (HDL-C) < 1.0 mmol/L; Hyperuricemia: Serum uric acid ⩾ 420μmol/L; Prediabetes: (7.0

mM > Fasting blood glucose (FBG) ⩾ 6.1 mM or 11.1 mM > 2 h Postprandial Blood Glucose (2 h PG) ⩾ 7.8 mM); Hypertension: systolic blood pressure (SBP) ⩾ 140 mmHg or diastolic blood pressure (DBP) ⩾ 90mmH.
